# Oligodendrocyte Egr2 Mediates the Beneficial Effect of Resocialization on the Social Ability of Socially Isolated Mice

**DOI:** 10.1007/s12264-025-01534-w

**Published:** 2025-11-06

**Authors:** Yanli Zhang, Sijia Chen, Shuying Zhang, Yue Li, Yimiao Wang, Min Cao, Yuxi Jin, Ze Wang, Shixin Ding, Ming Xiao

**Affiliations:** 1 Jiangsu Key Laboratory of Neurodegeneration, Nanjing Medical University, Nanjing, 211166 China; 2 Changzhou Medical Center, Nanjing Medical University, Changzhou, 213003 China; 3 Department of Geriatric Neurology, Nanjing Brain Hospital Affiliated to Nanjing Medical University, Nanjing, 210029 China

**Keywords:** Early social isolation, Social ability, Medial prefrontal cortex, Myelin plasticity, Resocialization

## Abstract

Early social isolation (SI) impairs social ability, which can be partially rescued after resocialization, but the underlying mechanisms have been rarely addressed. This study reported that adolescent SI mice resocialized with group housing (GH) mice, but not with SI mice, showed improved social behavior performances, increased myelination in the medial prefrontal cortex (mPFC), and upregulated Egr2 expression in oligodendrocytes (OLs). Specific down-regulation of OL Egr2 in GH companions or overexpression of OL Egr2 in SI peers abolished or rescued their repair effects on mPFC hypomyelination and social ability defects in SI mice, respectively. Furthermore, the improving effect of GH companions on OL Egr2 expression and myelinogenesis in the mPFC of SI mice was abolished when GH mice were treated with corticosterone. RNA-sequencing analysis showed that Egr2 enhanced myelination by inhibiting PDGFRα. Together, these results revealed that the Egr2/PDGFRα axis mediates distinct peer effects in rescuing SI-induced hypomyelination and social ability impairment.

**Supplementary Information:**

The online version contains supplementary material available at 10.1007/s12264-025-01534-w.

## Introduction

Social ability, encompassing social cooperation, behavior, and prosocial tendencies, is fundamental to the survival of social animals [1–3]. Early life experiences are crucial for establishing social behavior and abilities [4]. Notably, early social isolation (SI) not only impairs social ability but also increases the risk of neuropsychiatric disorders, such as depression and schizophrenia, in adulthood [5, 6]. Therefore, it is crucial to timely correct the negative consequences of early SI and explore the underlying mechanisms.

Resocialization, as a classic non-pharmacological intervention paradigm, enhances health conditions and behavioral performances; thus, it is utilized to delay aging and prevent Alzheimer’s disease (AD) and stress-associated mental illnesses [7–9]. In particular, mice that have experienced SI can change or adjust their behavioral norms and lifestyles after returning to the community environment [7]. The cognitive function and social skills of adopted or fostered orphans are superior to those of their peers [10]. Consistently, resocialization with group-housing (GH) mice improves SI-induced defects in social behavior among adolescent mice [11–13]. However, weaned mice that have been isolated during breeding exhibit impaired myelin formation in the medial prefrontal cortex (mPFC), a critical brain region for regulating social behavior, and this impairment cannot be reversed by cohabitating with other SI-experienced littermates [12]. These seemingly contradictory outcomes suggest that the restorative effects of resocialization on social ability impairment due to early SI are dependent upon the early experiences of their peers, although the underlying mechanism remains to be clarified.

In the present study, weaned male C57BL/6J mice underwent a 3-week SI, followed by a 4-week resocialization period with either GH or SI companions. We compared the effects of these two resocialization strategies on the cooperative, social, and prosocial behaviors of SI mice, as well as the changes in myelin sheath, synaptic ultrastructure, and related proteins in the mPFC. Additionally, early growth response 2 (Egr2) has been shown to regulate Schwann cell myelination and hindbrain development [14, 15]. Our recent study indicated that hypomyelination is associated with down-regulation of Egr2 expression in mPFC oligodendrocytes (OLs) of adolescent SI mice [11]. Based on this, we further investigated the regulatory role of Egr2 on mPFC myelination and social behavior of SI mice under the two different resocialization protocols. Finally, RNA-sequencing and gene manipulation strategies were employed to explore the mechanisms by which upstream and downstream factors of Egr2 could influence social ability defects in SI mice.

## Materials and Methods

### Animals and Experimental Design

Three-week-old male C57BL/6J mice were purchased from Experimental Animal Central of Nanjing Medical University. All these mice were bred at the Experimental Animal Center of Nanjing Medical University. They were randomly assigned to either the GH or SI group. In the GH group, 4 mice were housed in a single cage, whereas in the SI group, each cage contained only 1 mouse [16]. All mice were kept in specific-pathogen-free facilities with a consistent room temperature of 18–22°C, relative humidity of 30%–50%, a 12-h light/dark cycle, and had unlimited access to food and water. The animal experiments were carried out in compliance with the guidelines of the Institute of Experimental Animals at Nanjing Medical University (Approval number: IACUC: 2004013).

To establish the resocialized model [13], the SI mouse (*n* = 12) was housed with 3 GH mice in a single cage (totaling 12 cages), forming the resocialization with group house mice (Re-GH) group. For the resocialization with social isolation mice (Re-SI) group, 4 SI mice were group-housed per cage (totaling 3 cages, *n* = 12). In the GH group, 4 mice were maintained per cage (totaling 3 cages, *n* = 12), whereas in the SI group, each weaned mouse was consistently housed in a separate cage (totaling 12 cages, *n* = 12). The resocialization process spanned a duration of 4 weeks.

To assess the impact of Egr2 on social behavior and myelination in mPFC OLs during two modes of resocialization, adeno-associated virus serotype 9 (AAV9) with a specific 2',3'-cyclic nucleotide 3'-phosphodiesterase (CNP) promoter was used to overexpress Egr2 [AAV-CNP-Egr2 (AAV-CNPp-Egr2-EGFP-3Flag), 1 × 10^12^ vector genomes per milliliter (vg/mL)] or GFP [AAV-CNP-GFP (AAV-CNP-EGFP-3Flag), 1 × 10^12^ vg/mL] *via* stereotactic injection into the bilateral mPFC of 3-week-old SI mice (SI^CNP-Egr2^ or SI^GFP^). The weaned mice were kept in isolation for 3 weeks before being co-housed with GH^GFP^ (Re-GH^GFP^) (totaling 10 cages, *n* = 10), SI^CNP-Egr2^ (Re-SI^CNP-Egr2^) (totaling 10 cages, *n* = 10), and SI^GFP^ (Re-SI^GFP^) (totaling 10 cages, *n* = 10) mice for 4 weeks, respectively. To further confirm that Egr2 in OLs is necessary for resocialization, increasing cooperation defects caused by early SI, an *Egr2*-targeting shRNA under the CNP promoter [AAV-CNP-siEgr2 (AAV-CNPp-MCS-EGFP-Egr2), 1 × 10^12^ vg/mL] or AAV-CNP-GFP (AAV-CNP-RNAi-EGFP, 1 × 10^12^ vg/mL) was stereotaxically injected into the bilateral mPFC of 3-week-old GH mice (GH^CNP-siEgr2^ or GH^GFP^). Weaned mice were kept in isolation for 3 weeks before being co-housed with GH^GFP^ (Re-GH^GFP^) (totaling 10 cages, *n* = 10), GH^CNP-siEgr2^ (Re-GH^CNP-siEgr2^) (totaling 10 cages, *n* = 10), and SI^GFP^ (Re-SI^GFP^) (totaling 10 cages, *n* = 10) mice for 4 weeks, respectively. The viruses mentioned above were purchased from GeneChem, located in Shanghai, China. The sequence of siEgr2 was GCTGTACAGGAGATCTCTA [11].

To investigate whether serum corticosterone (Cort) plays a role in the behavioral and pathological effects of resocialization on SI mice, GH companions were intraperitoneally injected daily with Cort (#C2505, 30 mg/kg, Sigma-Aldrich, Missouri, USA) [17] or PBS for 4 weeks. Conversely, SI companions were intraperitoneally injected daily with metyrapone (Mety, #HY-B1232, 50 mg/kg, MCE, New Jersey, USA) or PBS for 4 weeks.

All the group mice mentioned above that had completed the modeling underwent the prosocial behavior test on the first day after the modeling. On the second day, the three-chamber test was conducted. The social cooperation test was conducted from day 3 to day 14. Following the sacrifice of the animals on day 15, histopathological analyses were conducted.

### Behavioral Tests

#### Social Cooperation Test

The social cooperative ability of mice was assessed using a cooperative drinking water device developed by our laboratory [18]. The device comprises a clear acrylic cage (60 cm × 40 cm × 35 cm) equipped with two photoelectric switches that work in tandem to control the flow of water droplets. Prior to testing, the mice were deprived of water for ~24 h. In the initial phase, a single subject mouse was permitted to freely explore the device for 5 min and learned to stand on the right side to activate the switch for drinking water when the other switch was activated. The training was conducted twice daily for 7 consecutive days. During the second phase, two trained mice were allowed to move freely for 10 min. The trial was conducted once daily for 5 days. The incubation period, frequency, and duration of drinking in both phases were recorded and analyzed.

#### Three-Chamber Test

The social behavior of the mice was assessed using a device consisting of three equidistant compartments (40 cm × 40 cm × 30 cm), separated by two square openings (8 cm × 8 cm), each of which was accessible [19]. Prior to the test, the subject mice were placed in the center compartment and allowed to freely explore for 5 min. During the sociality test, a sex- and age-matched mouse, referred to as stranger-1, was placed in a metal cage (8 cm × 8 cm) on one side of the compartment. The subject mice were then returned to the center compartment and permitted to explore freely among the compartments for another 5 min. The social novelty test commenced 1 h later, with a new sex- and age-matched mouse (stranger-2) placed in the metal cage on the opposite side. The subject mice were once again placed in the center compartment for 5 min of free exploration. The percentage of time spent by the mice in each compartment was recorded and subsequently analyzed.

#### Prosocial Behavior Test

For prosocial interaction, two unfamiliar mice from the same group were allowed to explore freely for 10 min in a new cage (dimensions: length 31 cm, width 22 cm, and height 15 cm) [20, 21]. The frequency and duration of social grooming and aggression were recorded and analyzed.

### Stereotaxic Bilateral Injection

Mice were anaesthetized by intraperitoneal (i.p.) injection of ketamine (80 mg/kg) and xylazine (8 mg/kg) in saline. Anesthetized mice that underwent skull exposure were immobilized in a stereotaxic device. The AAV9 virus was purchased from GeneChem (Shanghai, China) and injected into the bilateral mPFC using a 33-gauge syringe needle (Hamilton, Shanghai, China). The coordinates were +1.8 mm anterior-posterior relative to Bregma, −1.5 mm dorsal-ventral, ±0.4 mm medial-lateral, and the injection rate was 0.2 µL/min, and the cannula was left in place for 5 min following completion of the infusion [11, 22]. After the procedure, the mice were placed on a warmer pad until they awoke and then returned to their original cages.

### Tissue Preparation

Anesthetized mice were injected with 0.9% saline for 3 min, and half of the brain tissue was fixed overnight in 4% PFA. For electron microscopy (EM), the mPFC was dissected and fixed in 2.5% glutaraldehyde for a minimum of one week at 4°C. The mPFC samples were then stained with 70% ethanol containing 0.5% uranyl acetate for 1 h, dehydrated through a series of ethanol dilutions, cleaned with propylene oxide, embedded in Epon, and incubated at 60°C for 24 h. The tissues embedded in Epon were trimmed and subsequently sectioned into ultra-thin (70 nm) cross-sections using an ultra-microtome Leica EM UC7 (Leica Biosystems, Deer Park, Illinois, USA).

For immunofluorescent verification of viral injection sites and transduction efficiency, brain tissues underwent sequential dehydration in 20% and 30% sucrose solutions, followed by sectioning at 30 μm thickness (#Cryotome E, Thermo Fisher, Massachusetts, USA). For immunohistochemical and other immunofluorescent staining, the brain tissue was dehydrated using a gradient ethanol solution, coated with paraffin wax, and continuously sliced at a thickness of 5 μm using a paraffin microtome (#RM2245, Leica Biosystems). The remaining brain tissue was stored at −80°C for quantitative real-time polymerase chain reaction (qPCR) and Western blot analyses. Blood samples from resocialized model mice, which had been resocialized with GH mice that received intraperitoneal injections of Cort or PBS, were stored at 4°C overnight, centrifuged at 2,000 r/min for 20 min, and the serum was separated and collected for enzyme-linked immunosorbent assay (ELISA).

### Primary O4^+^ Immature OL Culture

As previously reported [16], the isolated cortex from newborn C57BL/6J mice (P6–P7) was cut into pieces in DMEM/F12 (#11320-033, Thermo Fisher) after the meninges and blood vessels were removed. The brain tissues were digested with papain (#a00324-0100, 20–30 U/mL, Sangon Biotech, Shanghai, China) and DNase I (#D4513, 2500 U, Sigma) at 37°C for 20 min. After being filtered through a 70 µm filter (#352340, BD Falcon, New Jersey, USA), the cells were resuspended in PBS containing anti-O4 microbeads (#130-094-543, Miltenyi Biotec, Cologne, Germany) and incubated at 4°C for 15 min. The beads were then captured using a column (#130-042-201, Miltenyi Biotec) to enrich O4^+^ cells. O4^+^ immature OLs were cultured in Neurobasal medium supplemented with 1% N2 (#17502-048, Thermo Fisher), 2% B27 (#17504044, Gibco Thermo Fisher), 1% penicillin-streptomycin (#15140-122, Gibco Thermo Fisher), and 40 ng/mL PDGF-AA (#1055-AA-050, R&D Systems, Minnesota, USA) at a density of (0.9–1.5) × 10^4^ cells/cm^2^. The medium was entirely replaced after 24 h, and subsequently, half of the medium was replaced every two days for a period of 6–7 days. To facilitate the maturation of OLs, they were cultured in a medium containing 1% N2, 2% B27, 1% penicillin-streptomycin, 50 µg/mL insulin (#I-6634, Sigma), 40 ng/mL triiodothyronine (#T2877, Sigma), and 1 ng/mL ciliary neurotrophic factor (#557-NT, R&D Systems). After a further 2 days of culture, the *Egr2* interference plasmid was transfected, and the cells were harvested 48 h later for RNA-seq analysis.

### Mouse Oligodendrocyte Precursor Cell (mOPC) Line Culture

mOPCs were cultured in DMEM/high-glucose supplemented with 10% fetal bovine serum (#10099158, Gibco Thermo Fisher) and 1% penicillin-streptomycin. The medium was refreshed every 1 to 2 days.

### Primary OLs and mOPC Treatment

For primary OLs, small interfering RNAs (siRNAs) targeting *Egr2* and *Pdgfrα*, as well as a scrambled siRNA, were procured from RiboBio (Guangzhou, China). The sequence for siEgr2 was GCTGTACAGGAGATCTCTA, and for siPdgfrα, it was TATAATGGCAGAATCATCATT. Overexpression plasmids for *Pdgfrα* or *Egr2* (pcDNA3.1-PDGFRα or pcDNA3.1-Egr2), the pcDNA3.1 vector, the PGL3-basic vector, and the PDGFRα luciferase reporter (pGL3-PDGFRα) were obtained from GenScript. Cells were transfected and harvested after 48 h for the dual-luciferase reporter gene assay. For mouse OPCs, transfection was carried out following 24 h of incubation, as per the manufacturer’s guidelines, and the cells were subsequently utilized for chromatin immunoprecipitation (ChIP) assays.

### Immunostaining

For immunohistochemistry, the deparaffinized brain tissue was blocked with goat serum at room temperature for 1 h and then incubated with primary antibodies as follows: anti-postsynaptic density protein 95 (PSD-95) (#ab18258, 1:200, Abcam, Cambridge, UK), anti-synaptophysin (SYP) (#ab32127, 1:500, Abcam), and anti-myelin basic protein (MBP) (#ab7349, 1:400, Abcam) overnight at 4°C. Horseradish peroxidase secondary antibodies (#ZB-2301, #ZB-2307, ZSGB-BIO, Beijing, China) were incubated at 37°C for 1 h, followed by visualization with diaminobenzidine (#91-95-2, Sigma-Aldrich). For immunofluorescence, the deparaffinized brain tissue and cultured cells were blocked with 5% bovine serum albumin containing 0.03% Triton X at room temperature for 1 h, and subsequently incubated with primary antibody overnight at 4°C. The primary antibodies used were anti-MBP (#ab7349, 1:400, Abcam), anti-PDGFRα (#ab203491, 1:200, Abcam), anti-Egr2 (#NB100-92327, 1:200, Novus, Missouri, USA), anti-O4 (#MAB1326, 1:200, R&D Systems), anti-GR (#4161s, 1:200, CST, Boston, USA), anti-mineralocorticoid receptor (#bs-1850R, 1:200, MR, Bioss, Beijing, China), and anti-NeuN (#ab177487, 1:200, Abcam). Following a wash with PBS, the tissues were incubated with corresponding fluorescent probe-conjugated secondary antibodies (#A21202, #A31572, #A21206, #A31571, and #A31570, 1:1,000, Thermo Fisher) at room temperature for 2 h. Nuclei were stained with DAPI (#R37606, 1:1,000, Life Technologies, California, USA). Images were captured using the LSM710 confocal microscope (LSM710, Zeiss, Jena, Germany).

### ELISA

The ELISA (Nanjing Xin Fan Biology, China) was utilized to detect Cort levels. After the ELISA kit was allowed to equilibrate at room temperature for 1 h, the samples (50 μL) and horseradish peroxidase conjugated reagent (100 μL) were added and incubated at 37°C for 1 h. Subsequently, the chromogen solutions A (50 μL) and B (50 μL) were added and incubated at 37°C for 15 min, followed by the addition of the termination solution (50 μL). The optical density (OD) was measured at 450 nm within 15 min using a microplate reader (Multiskan FC, Thermo Fisher).

### Western Blot

An equal amount of protein from brain tissue and primary OLs was separated using sodium dodecyl sulfate polyacrylamide gel electrophoresis (SDS-PAGE) on a 12% gel and subsequently transferred to a polyvinylidene fluoride membrane (#ISEQ00010, Millipore, Massachusetts, USA). The membrane was blocked with Tris-buffered saline containing 0.1% Tween-20 (TBST) and 5% skim milk for 1 h, followed by overnight incubation at 4°C with the following primary antibodies: anti-Egr2 (#sc-293195, 1:1,000, Santa Cruz, California, USA), anti-MBP (#ab7349, 1:1,000, Abcam), anti-PSD-95 (#ab18258, 1:1,000, Abcam), anti-PDGFRα (#MAB1326, 1:100, R&D Systems), anti-SYP (#ab32127, 1:2,000, Abcam), and anti-GAPDH (#60004-1-Ig, 1:3,000, Proteintech, Wuhan, China). The bands were subsequently incubated with horseradish peroxidase-conjugated secondary antibodies (ZSGB-BIO, Beijing, China) at 25°C for 1 h and visualized using the ECL Plus detection system (ImageQuant LAS4000 mini, Cytiva, Shanghai, China). GAPDH served as an internal reference, and ImageJ (version 1.6.0, NIH) was utilized for quantification. The uncropped Western blot data are presented in the Supplementary Materials.

### qPCR

TRIzol (#9109, Takara, Kyoto, Japan) was used to extract the total RNA from the mPFC or primary OLs. The qPCR was conducted using the SYBR Green PCR master mix on an ABI Step One Plus Real-Time PCR System (Applied Biosystems, Foster City, USA). The cDNAs were synthesized from 1 µg of total RNA using the Maxima First Strand Synthesis Kit for qPCR (#RR047B, Takara). The qPCR involved amplifying cDNA for 40 cycles with the SYBR Green PCR master mix. The mRNA levels were normalized against GAPDH. The primers are listed in Table S1. Relative quantification of the target genes was performed using the ΔΔCt method.

### Dual-Luciferase Reporter Gene Assay

The binding relationship between Egr2 and PDGFRα was first predicted by JASPAR (http://jaspar.genereg.net/), and subsequently validated through a dual-luciferase reporter gene assay (#E1910, Promega, Wisconsin, USA). A 0.05 μg PDGFRα luciferase reporter plasmid and a 0.2 μg Egr2 overexpression plasmid were co-transfected into mOPCs. After 48 h, the cell lysates were utilized to measure their relative luciferase activity. The luciferase activity was evaluated using a microplate reader (Multiskan FC, Thermo Fisher), with the firefly luciferase activity normalized to the Renilla luciferase activity.

### ChIP Analysis

We utilized a Magna ChIP Kit (#17-10085, Millipore) for ChIP analysis [23]. Briefly, mOPCs were crosslinked with 1% formaldehyde for 10 min at room temperature; the reaction was then quenched with 0.125 mol/L glycine for 5 min. The fixed cells were lysed with 0.5 mL of Cell Lysis Buffer and 2.5 μL of Protease Inhibitor Cocktail II at 4°C for 15 min. Chromatin was resuspended in 0.5 mL of Nuclear Lysis Buffer at 4°C. The nucleus was disrupted by one pulse of a bioruptor with high output, resulting in fragments ranging from 200 bp to 1,000 bp. The samples were then incubated with anti-Egr2 (#sc-293195, Santa Cruz) or anti-RNA polymerase II (#17-10085, Millipore, Massachusetts, USA) antibodies. Chromatin was incubated overnight at 4°C, followed by the addition of protein A/G magnetic beads to the reaction for an additional hour. The magnetic beads were washed, and the chromatin was eluted and reversed. Subsequently, the chromatin was treated with proteinase K and purified using a Gel Extraction Kit (D0043M, BeyoMag, Shanghai, China). The ChIP DNA was then used for ChIP-qPCR. The PCR primers are listed in Table S2.

### RNA-seq and Data Analysis

The differentiated OLs were treated with siEgr2 for 48 h to knock out *Egr2*. Total RNA was extracted using TRIzol (#9109, Takara), and RNA-seq was performed. The RNA library was constructed using the Illumina platform according to the manufacturer’s instructions; adapters were ligated to each end of the RNAs, and subsequently reverse transcribed to create single-stranded cDNA, which was sequenced on the Illumina platform with a read length of 50 bp. Stringent criteria were set to determine significantly dysregulated genes (unknown genes have been excluded): an adjusted *P-*value <0.05 and log_2_fold change (FC) >0.5. OLs treated with or without siEgr2 were analyzed as two independent datasets. All RNA-seq datasets are available at GEO (https://www.ncbi.nlm.nih.gov/geo/). The RNA-seq of differentiated OLs was treated with siEgr2 for 48 h (GSE272732), and mPFC tissues of P59 GH or SI mice (GSE162343) were analyzed by R (v4.3.1). The R packages clusterProfiler (v4.8.3) and msigdbr (v7.5.1) were used to enrich the gene sets. Bubble plot, ridge plot, and Gene Set Enrichment Analysis (GSEA) plot were performed using the packages ggplot2 (v3.4.3) and enrichplot (v1.20.1).

### Protein–Protein Interaction (PPI) Analysis

PPI network analysis was conducted to predict the relationship between Egr2 and genes involved in the top-ranked functions enriched by GO analysis, utilizing the STRING v12.0 database (retrieved and downloaded on April 2, 2024) (STRING, https://www.string-db.org).

### Image Analysis

Immunohistochemical and immunofluorescent images were captured using the Zeiss LSM880 confocal microscope. The immunohistochemical staining of MBP, SYP, and PSD-95 in the mouse mPFC was analyzed by mean integrated optical density (MIOD). For immunofluorescence, the positive area of MBP^+^ or Egr2^+^ in the corresponding image was measured using gray threshold analysis. The cells of O4^+^, Egr2^+^, Egr2^+^NeuN^+^/NeuN^+^, O4^+^Egr2^+^/O4^+^, PDGFRα^+^, and Egr2^+^PDGFRα^+^/PDGFRα^+^ in the mPFC were counted as the number of positive cells per mm^2^, respectively. The “Sholl” analysis plugin in ImageJ was utilized to measure the process complexity of OLs. The measurement begins at a diameter of 0.05 μm and continues with an interval of 0.05 μm [11]. Three sections were selected from each mouse, with the average values derived from 5 to 8 mice per group. Additionally, the average values of immunohistochemistry and immunofluorescence for each group were considered.

EM images were utilized to detect OLs and synaptic integrity using a FEI Tecnai G2 electron microscope (FEI Company, Oregon, USA) at an acceleration voltage of 120 kV. In the EM images, OLs display a fusiform morphology with thin margins of perinuclear cytoplasm containing microtubules, but lacking intermediate filaments or glycogen particles [24]. To calculate the total nuclear area of each cell, heterochromatin was selected using the threshold tool, defined qualitatively as a gray density of 100 or greater on a 256 gray scale, and the percentage of the total nuclear area was subsequently calculated. For myelin analysis, 25 to 40 myelinated axons were assessed per mouse. The *g*-ratio was determined by dividing the diameter of the axon by the diameter of the entire myelin sheath. Synaptic integrity was analyzed by measuring the postsynaptic dense zone thickness, synaptic gap width, active zone length, and synaptic curvature. An average of 10 measurements of the synaptic gap width was taken as a reading for each synapse, with the end of the synapse defined as the end of the electron-dense PSD. The length of the active region is measured, and then the synaptic curvature is calculated using the ratio of the arc length of the active region to its corresponding chord length. To prevent bias, the image analysis was conducted by an individual blind to the experimental conditions.

### Statistical Analysis

GraphPad Prism 8.0 (GraphPad Software, California, USA) was utilized for statistical analysis. Cooperative behavior data were analyzed using repeated measures analysis of variance (ANOVA). A two-tailed Student’s *t*-test was employed for comparisons between two groups. When comparing three or more groups, a one-way, two-way ANOVA, or three-way ANOVA followed by a Tukey’s *post hoc* test was applied. Data are presented as the mean ± SEM, and* P* <0.05 is deemed statistically significant.

## Results

### Improved Social Ability and mPFC Myelination in Early SI Mice After Resocialization with GH Rather than SI Companions

**Fig. 1:**
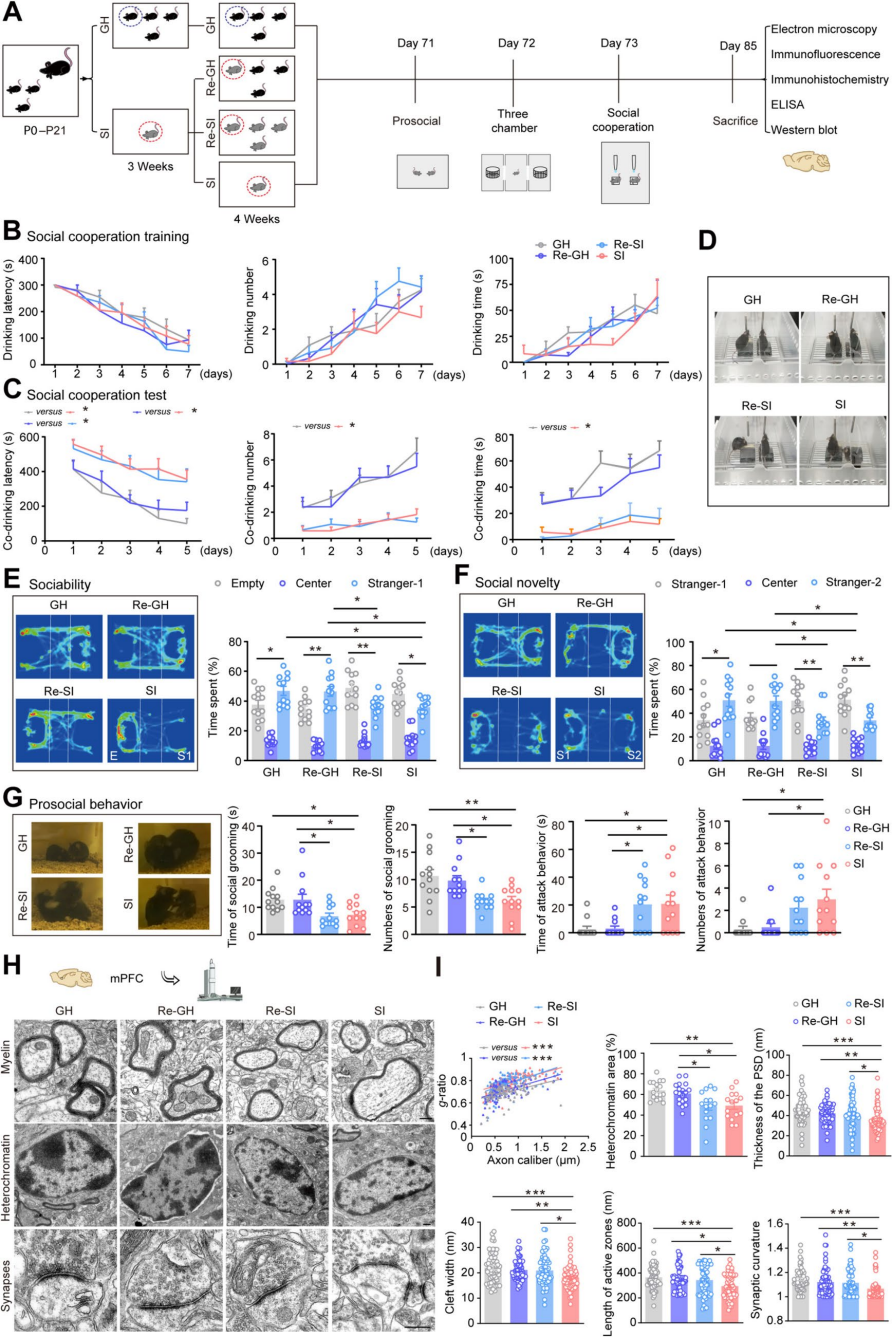
Resocialization of GH mice, but not SI mice, improved the decline in social competence and myelin damage induced by SI. **A** Schematic diagram showing the timeline of the experimental design for two resocialization modes of SI mice. **B**, **C** Bar graphs showing the drinking latency, drinking number, and drinking time of mice during the training period (**B**) and their cooperation performance during the testing period (**C**) of the cooperation drinking test (*n* = 12). **D** Representative images showing the cooperation drinking test period of mice. **E**, **F** Heat map and graph showing the sociability (**E**) and social novelty (**F**) of mice evaluated by the three-chamber test (*n* = 12). **G** Representative images and corresponding chart showing the time and number of social grooming and attacks during the prosocial behavior in mice (*n* = 12). **H** Representative electron microscopy (EM) images showing myelin, OL nuclear heterochromatin, and synaptic morphology in the mPFC of mice. Scale bars, 500 nm. **I** Scatter plot of *g*-ratios with linear least squares fitting (GH: 106 axons; Re-GH: 107 axons; Re-SI: 104 axons; SI: 110 axons). Graph showing the area percentage of OL nuclear heterochromatin (GH: 17 nuclei; Re-GH: 19 nuclei; Re-SI: 15 nuclei; SI: 15 nuclei) and quantification of PSD, synaptic cleft width, length of the active zones, and synaptic curvature (GH: 64 synapses; Re-GH: 67 synapses; Re-SI: 59 synapses; SI: 64 synapses) (*n* = 6). Data are presented as the mean ± SEM. *P <0.05, **P <0.01, ***P <0.001. Data in **B** and **C** were analyzed by repeated-measures ANOVA with *post hoc* Student-Newman-Keuls test, in **E** and **F** were analyzed by three-way ANOVA followed by Tukey’s multiple comparisons test, and in **G** and **I** were analyzed by two-way ANOVA followed by Tukey’s *post hoc* test. mPFC: medial prefrontal cortex; GH: group housing; Re: resocialization; SI: social isolation.

To confirm that partners’ early lifestyle influences the ameliorative effect of social ability impairment caused by early SI, weaned mice isolated for 3 weeks were resocialized with GH mice and SI mice for another 4 weeks, followed by behavior and neuropathological analyses (Fig. 1A). A water-reward test, established by our laboratory for evaluating cooperative behavior of mice [18], showed that during the training stage, there were no significant changes in the latency, number, and total time of drinking water among the Re-GH, Re-SI, SI, and GH groups (Fig. 1B). However, during the testing phase, the SI group exhibited a longer latency to co-drink and fewer instances of co-drinking, along with a shorter total co-drinking duration compared to the GH group (Fig. 1C, D). Additionally, when compared to the SI group, the Re-GH group demonstrated a reduced latency to co-drink water, and an increase in both the frequency and total duration of co-drinking (Fig. 1C, D). There were no significant differences observed between the SI mice and the Re-SI mice (Fig. 1C, D). In alignment with these findings, the three-chamber test revealed that the SI group had lower sociability and spent less time in the stranger-2 chamber during the social novelty test compared to the GH group (Fig. 1E, F). The sociability and social novelty were significantly increased in the Re-GH group, but not in the Re-SI group, when compared with those in the SI group (Fig. 1E, F). Additionally, SI mice exhibited a higher number of attacks and a lower number and less time spent on mutual grooming. In the Re-GH group, mutual grooming increased and aggressive behavior decreased, but this improvement was not observed in the Re-SI mice (Fig. 1G). Collectively, these results indicated that only resocialization with GH mice ameliorated the social ability defects of SI mice.

Previous studies indicated that the mPFC is a key brain region involved in early SI [11, 25, 26]. The results showed that SI mice had decreases in myelin thickness, OL nuclear heterochromatin area, and MBP levels in the mPFC, which were corrected by resocialization with GH mice, but not with SI mice (Fig. 1H, I and Fig. S1A**–**C). However, both resocialization strategies significantly improved the synaptic parameters in SI mice, including SYP and PSD-95 levels (Fig. 1H, I and Fig. S1C**–**E).

### The mPFC OL Egr2 Is Essential for Resocialization to Enhance Social Ability and Myelination in SI Mice

**Fig. 2:**
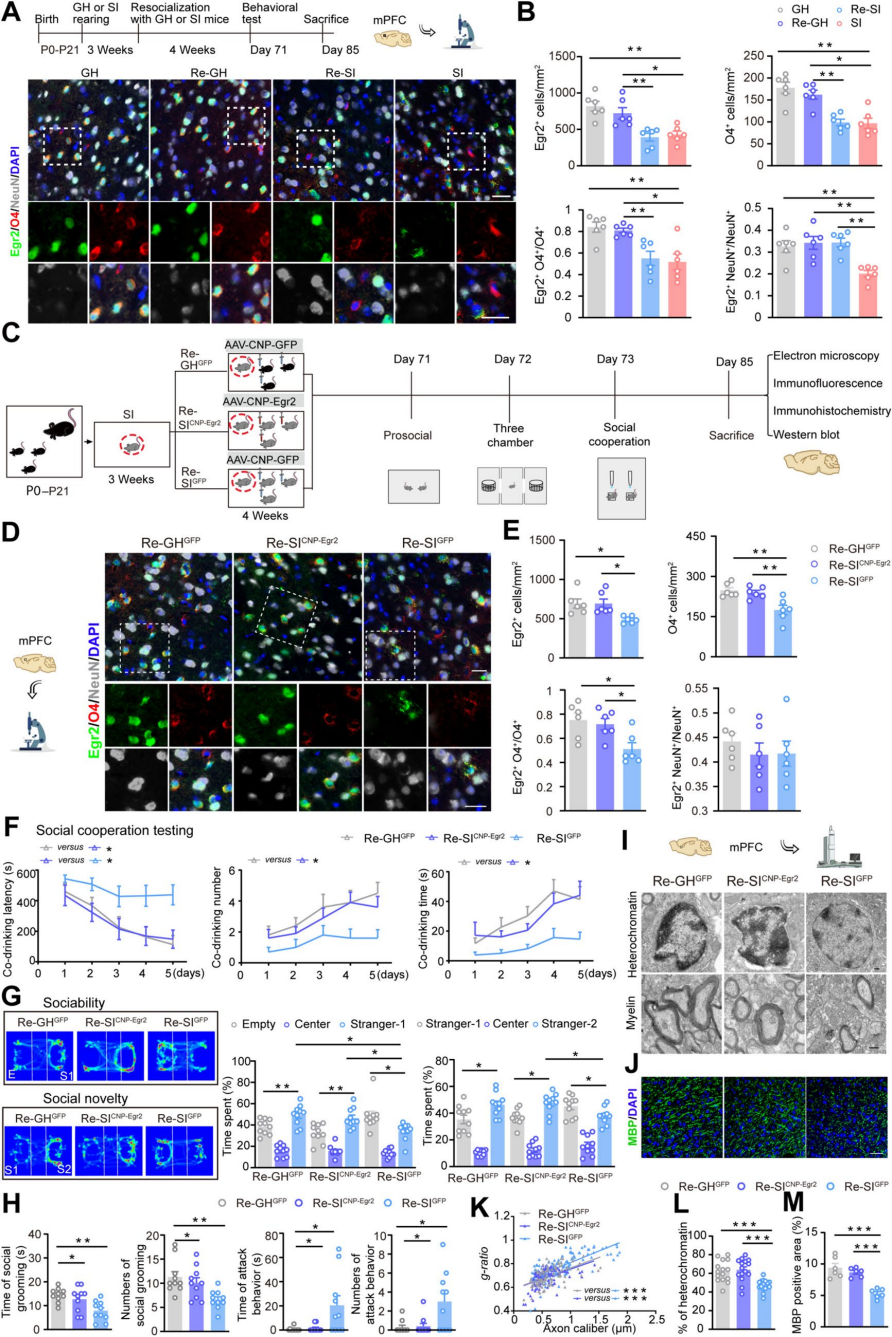
Resocialization of SI mice with Egr2 overexpression in mPFC OLs improved their social abilities and alleviated myelin damage. **A**, **B** Representative immunofluorescence images and corresponding graph showing Egr2^+^ (green), O4^+^ (red), O4^+^ Egr2^+^/O4^+^, neuronal nuclei (NeuN^+^; gray), and Egr2^+^/NeuN^+^ expression in the mPFC of SI mice resocialized with GH or SI mice (*n* = 6). Scale bars, 50 μm. **C** Schematic diagram showing the timeline of the experimental design for overexpressing Egr2 in mPFC OLs of SI mice. **D**, **E** Representative immunofluorescence images and corresponding graph showing Egr2^+^ (green), O4^+^ (red), O4^+^ Egr2^+^/O4^+^, NeuN^+^ (gray) Egr2^+^/NeuN^+^ expression in the mPFC of mice (*n* = 6). Scale bars, 50 μm. **F** Bar graphs showing the co-drinking latency (left), co-drinking number (middle), and co-drinking time (right) of mice during the social cooperation testing period (*n* = 10). **G** Heat map and bar graphs showing the sociability and social novelty of mice evaluated by the three-chamber test (*n* = 10). **H** Chart showing the time and number of social grooming and attacks during the prosocial behavior in mice (*n* = 10). **I** Representative EM images showing OL nuclear heterochromatin and myelin in the mPFC of mice. Scale bars, 500 nm. **J** Representative immunofluorescence images showing MBP (green) expression in the mPFC of mice. Scale bars, 50 μm. **K** Scatter plot of *g*-ratios with linear least squares fitting (Re-GH^GFP^: 202 axons; Re-SI^CNP-Egr2^: 190 axons; Re-SI^GFP^: 201 axons) (*n* = 6). **L** Graph showing the area percentage of OL nuclear heterochromatin (Re-GH^GFP^: 16 nuclei; Re-SI^CNP-Egr2^: 15 nuclei; Re-SI^GFP^: 16 nuclei) (*n* = 6). **M** Graph showing MBP expression in the mPFC of mice (*n* = 6). Data are presented as the mean ± SEM. *P <0.05, **P <0.01, ***P <0.001. Data in **B** and **G** were analyzed by Two-way ANOVA followed by Tukey’s *post hoc* test, data in **F** were analyzed by repeated-measures ANOVA with *post hoc* Student-Newman-Keuls test, and in others were analyzed by one-way ANOVA followed by Tukey’s *post hoc* test. mPFC: medial prefrontal cortex; GH: group housing; Re: resocialization; SI: social isolation.

Egr2 has been shown to be essential for myelination in the postnatal brain [11, 27]. The up-regulation of Egr2 expression in the mPFC can improve social behaviors of adolescent SI mice [11]. Here, we investigated the effects of different resocialization methods on Egr2 expression in the mPFC of SI mice. Immunohistochemistry revealed that mPFC Egr2 levels in SI mice were significantly reduced compared with those in GH mice. Egr2 levels were markedly elevated in the Re-GH group, but not in the Re-SI group (Fig. S1F). Moreover, double immunofluorescent staining revealed that Egr2 expression levels in OLs and neurons were reversed in Re-GH mice, whereas in Re-SI mice, only neuronal Egr2 was increased (Fig. 2A, B).

We established an AAV that overexpresses Egr2 under the control of the CNP promoter to investigate the role of Egr2 expression by OLs in remyelination and the repair of hypomyelination and social ability deficits resulting from early SI (Fig. S2A**–**D). Weanling SI mice were co-housed with SI littermates that had been injected with AAV-CNP-Egr2 into the bilateral mPFC for a duration of 4 weeks (Fig. 2C). Immunofluorescence confirmed the accuracy and efficacy of the injection site (Fig. S3A**–**C). The Re-SI^CNP-Egr2^ group exhibited a higher number of O4^+^Egr2^+^ cells in the mPFC compared to the Re-SI^GFP^ group, yet the number of Egr2^+^ neurons remained unchanged (Fig. 2D, E). The Re-SI^CNP-Egr2^ group demonstrated superior social behavioral performance compared to the Re-SI^GFP^ group (Fig. 2F–H and Fig. S3D). Myelin thickness, the area of OL nuclear heterochromatin, and MBP expression levels in the mPFC were greater in the Re-SI^CNP-Egr2^ group than in the Re-SI^GFP^ mice (Fig. 2I–M and Fig. S3E). However, the mPFC synaptic ultrastructure and the levels of SYP and PSD-95 did not show significant changes (Fig. S3E**–**I). These findings suggest that resocialization with SI companions that overexpress Egr2 in mPFC OLs can restore social ability and repair myelin damage in SI mice.

To further confirm that OL Egr2 is necessary for resocialization and improving social behavior impairments caused by early SI, we injected CNP-siEgr2 into the bilateral mPFC of GH mice. Weaned mice were kept alone for 3 weeks and then co-housed with GH^CNP-siEgr2^ mice and GH^GFP^ mice for 4 weeks, respectively, followed by behavioral and pathological analysis (Fig. S4A). Immunofluorescence confirmed the accuracy and effectiveness of the virus injection site (Fig. S4B**–**D). The behavioral results indicated that, in comparison to Re-GH^GFP^ mice, the Re-GH^CNP-siEgr2^ group exhibited deficiencies in social cooperation, sociability, social novelty, and grooming behaviors (Fig. S4E**–**H). In agreement with these findings, Re-GH^CNP-siEgr2^ mice showed reduced myelin thickness, decreased heterochromatin area in OL nuclei, and lower MBP levels in the mPFC when compared to Re-GH^GFP^ mice. However, there were no observed changes in synaptic ultrastructure or in the levels of SYP and PSD-95, relative to Re-GH^GFP^ mice (Fig. S5A**–**K). In summary, these outcomes suggest that OL Egr2 is essential for myelination in the mPFC and for the social ability deficits seen in SI mice during resocialization.

### Cort Regulated Egr2 Expression in mPFC OLs and Social Behavior in SI Mice Following Resocialization

**Fig. 3:**
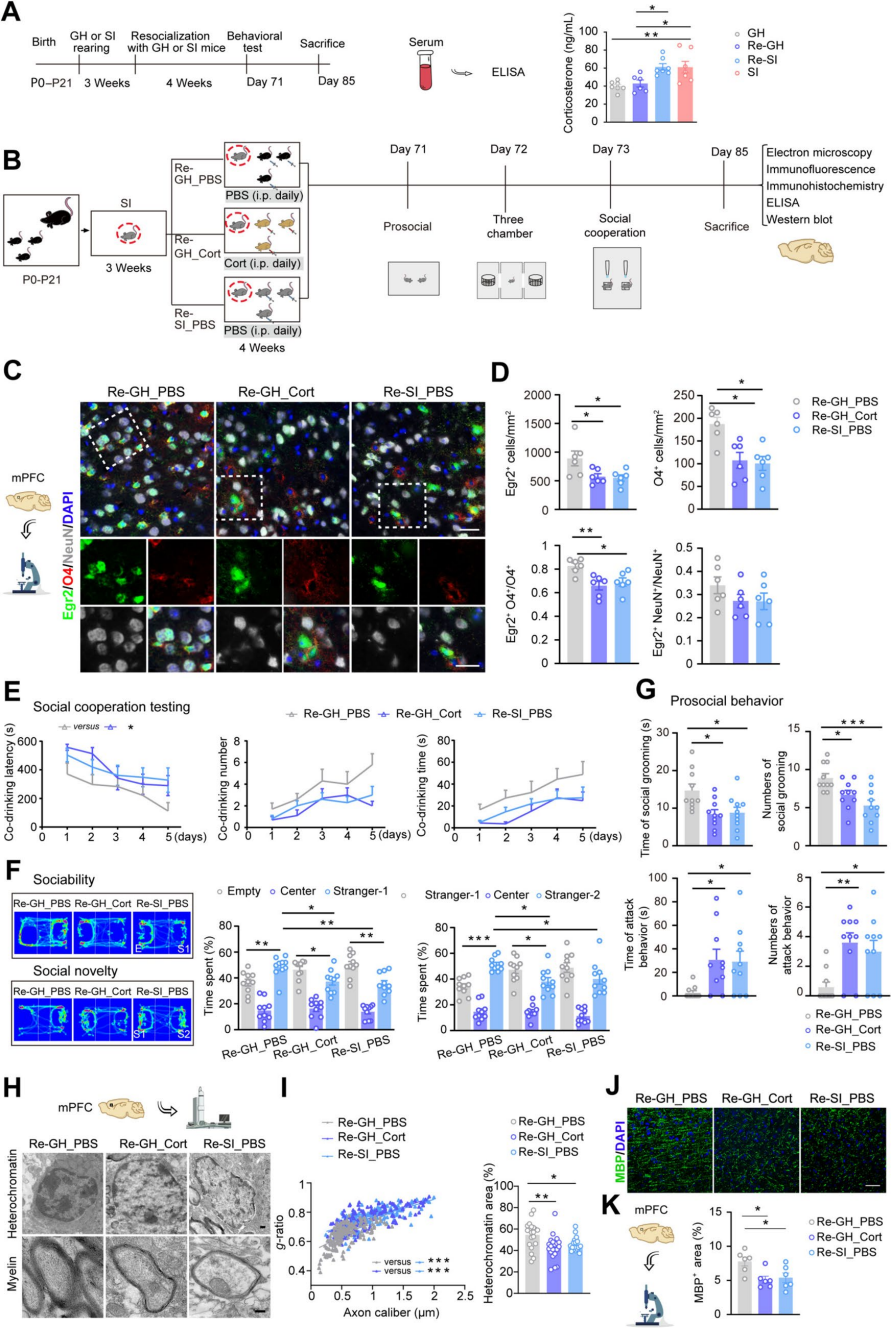
Exposure to Cort eliminated the enhancing effect of resocialization on social ability and myelin in SI mice. **A** ELISA results showing Cort levels in the serum of SI mice resocialized with GH or SI mice (*n* = 6–7). **B** A schematic diagram showing the timeline of the experimental design for SI mice housing with GH mice intraperitoneally injected with Cort. **C**, **D** Representative immunofluorescence images and the corresponding graph showing Egr2^+^ (green), O4^+^ (red), O4^+^ Egr2^+^/O4^+^, NeuN^+^ (gray) Egr2^+^/NeuN^+^ expression in the mPFC of SI mice resocialized with GH mice with Cort treatment (*n* = 6). Scale bars, 50 μm. **E** Bar graphs showing the co-drinking latency, co-drinking number, and co-drinking time of mice in the testing stage (*n* = 10). **F** Heat map and bar graphs showing the sociability and social novelty of mice evaluated by the three-chamber test (*n* = 10). **G** Chart showing the time and number of social grooming and attacks during the prosocial behavior in mice (*n* = 10). **H** Representative EM images showing OL nuclear heterochromatin and myelin in the mPFC of mice. Scale bars, 500 nm. **I** Scatter plot of *g*-ratios with linear least squares fitting (Re-GH_PBS: 142 axons; Re-SI_Mety: 131 axons; Re-SI_PBS: 155 axons) (*n* = 6). Graph showing the area percentage of OL nuclear heterochromatin in (Re-GH_PBS: 21 nuclei; Re-SI_Mety: 22 nuclei; Re-SI_PBS: 21 nuclei) (*n* = 6). **J**, **K** Representative immunofluorescence images and corresponding graph showing MBP (green) expression in the mPFC of mice (*n* = 6). Scale bar, 50 μm. Data are presented as the mean ± SEM. *P <0.05, **P <0.01, ***P <0.001. Data in **E** were analyzed by repeated-measures ANOVA with *post hoc* Student-Newman–Keuls test, data in **F** were analyzed by two-way ANOVA followed by Tukey’s *post hoc* test, and others were analyzed by one-way ANOVA followed by Tukey’s *post hoc* test. GH: group housing; Re: resocialization; SI: social isolation; mPFC: medial prefrontal cortex; Cort: corticosterone; Mety: metyrapone.

Next, we compared the RNA sequencing data of the mPFC between SI mice and GH mice (GSE162343) to explore the upstream mechanisms regulating Egr2 expression. GSEA revealed the most significant alterations in the corticotropin-releasing factor (CRF) signaling pathway (Fig. S6A**–**D). CRF is the initiating hormone of the hypothalamic-pituitary-adrenal (HPA) axis adaptive stress response [28]. Our recent study revealed that Cort treatment suppressed Egr2 transcriptional and protein levels in cultured primary OLs [11]. Consequently, we assessed the serum Cort levels in mice using ELISA (Fig. 3A). SI mice exhibited elevated serum Cort levels compared to GH mice. However, serum Cort levels were notably decreased in Re-GH mice, but not in Re-SI mice (Fig. 3A). And we further found that in resocialization with GH mice, but not in SI mice, the expression of GR and MR in oligodendrocytes was improved in the mPFC (Fig. S7A, B). These findings suggest that the varying effects on behavior and pathology observed in SI and GH mice post-resocialization may be associated with serum Cort levels.

To confirm this hypothesis, daily intraperitoneal injections of Cort in SI mice’s GH companions were administered over a period of 4 weeks (Fig. 3B). ELISA results indicated that Cort supplementation increased the serum Cort levels in GH mice (Fig. S8A, B). Moreover, Cort treatment of GH companions counteracted the reduction in serum Cort levels in SI mice following resocialization (Fig. S8C). Immunofluorescence results demonstrated that resocialization of SI mice with Cort-treated GH companions negated the improvement in Egr2 levels in mPFC OLs (Fig. 3C, D). The Re-GH_Cort group exhibited impaired social cooperation, sociability, social novelty, and grooming behaviors compared to the Re-GH_PBS group (Fig. 3E–G and Fig. S8D). Histopathological results indicated that myelin thickness, heterochromatin area of oligodendrocyte nuclei, and MBP-positive area were also reduced in the mPFC of the Re-GH_Cort group (Fig. 3H–K and Fig. S8E). However, the ultrastructure of mPFC synapses and the expression levels of SYP and PSD-95 showed no significant differences among the three groups of mice (Fig. S8E**–**I).

To confirm the influence of Cort on the behavior and pathology of isolated mice during resocialization, weaned SI mice were co-housed with other SI companions treated with daily intraperitoneal injections of Mety, an antagonist of Cort, for 4 weeks (Fig. S9A). ELISA revealed that the serum Cort level in the SI_Mety group mice was significantly decreased compared to that in the SI_PBS group mice (Fig. S9B). Additionally, the serum Cort level in the Re-SI_Mety group mice was significantly lower than that in the Re-SI_PBS group mice (Fig. S9C). Immunofluorescence results indicated that resocialization of SI mice with Mety-treated SI companions led to increased Egr2 levels in mPFC OLs (Fig. S9D). Behavioral outcomes revealed that, compared to the Re-SI_PBS group, Re-SI_Mety group mice exhibited enhancements in social behaviors (Fig. S9E**–**H). In line with these findings, myelin thickness, OL nuclear heterochromatin area, and MBP levels in the mPFC were elevated in Re-SI_Mety group mice, yet synaptic ultrastructure and the levels of SYP and PSD-95 remained unchanged (Fig. S10A**–**K). Collectively, these results suggest that Cort inhibits Egr2 expression in mPFC OLs post-resocialization, thereby impairing social behavior in SI mice.

### Egr2 Regulated mPFC Myelin Development by Inhibiting the Expression of Platelet-Derived Growth Factor Receptor Alpha (PDGFRα)

**Fig. 4:**
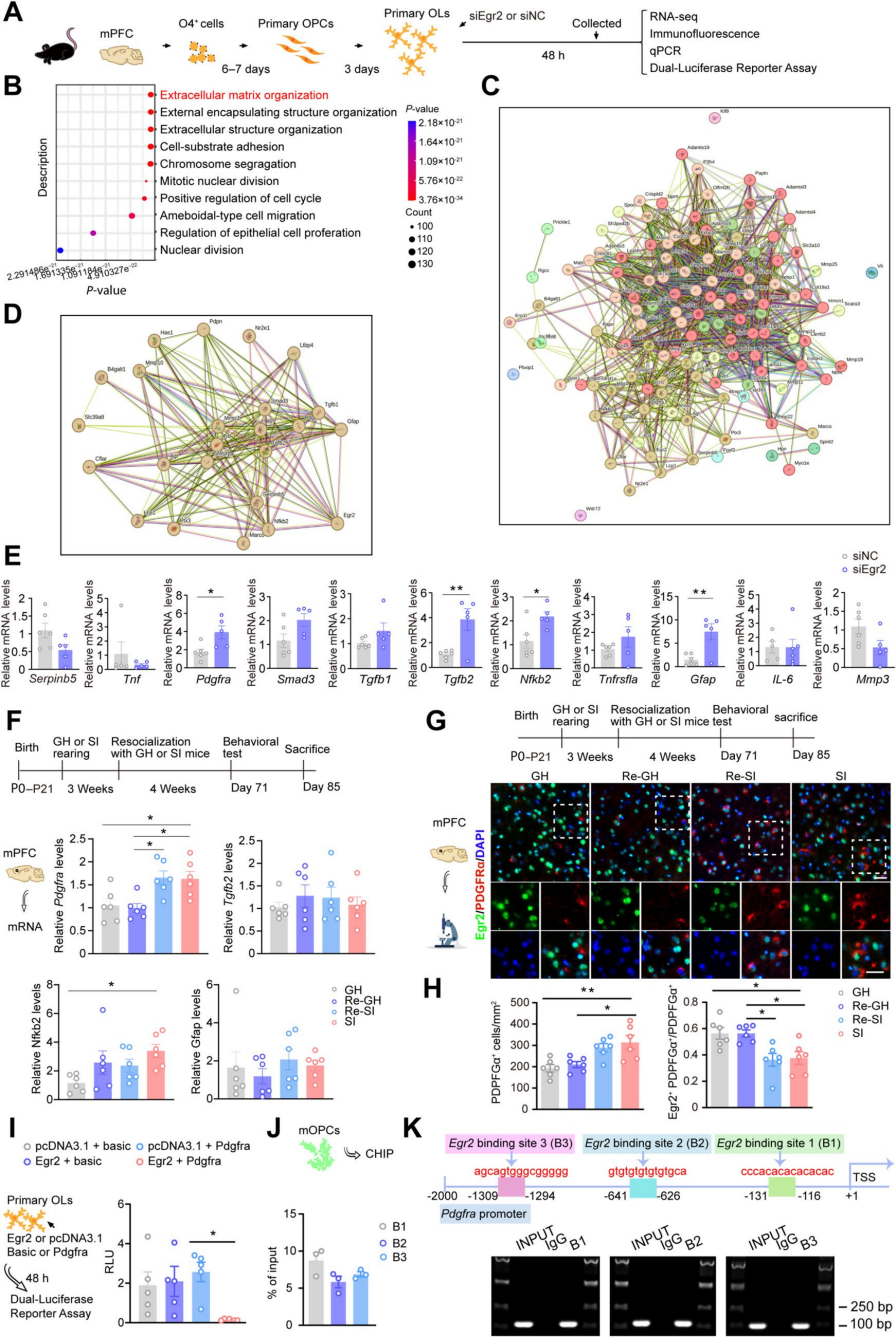
Egr2 promoted the differentiation and maturation of OLs by inhibiting the expression of PDGFRα. **A**, **B** Top 10 GO enrichment analysis of primary OLs down-regulated Egr2 levels. **C**, **D** PPI network showing the relationship of Egr2 and all genes in extracellular matrix organization. **E** qPCR showing the mRNA levels of the direct genes linked to Egr2 in the PPI network (*n* = 6). **F** qPCR detecting the expression of *Pdgfra*, *Tgfb2*, *Gfap*, and *Nfkb* in the mPFC of SI mice under different resocialization methods (*n* = 6). **G**, **H** Representative immunofluorescence images and corresponding graph showing PDGFRα^+^ (red) and Egr2^+^ (green) PDGFRα^+^/PDGFRα^+^ expression in the mPFC of SI mice resocialization with GH or SI mice (*n* = 6). Scale bars, 50 μm. **I** PDGFRα promoter-driven luciferase activities in primary OLs with the indicated transfections (*n* = 5). Relative light unit (RLU) is a relative measurement of the amount of light produced in a sample. **J**, **K** ChIP-qPCR revealing binding between *Egr2* and the promoter region of PDGFRα in mOPCs. Data are presented as the mean ± SEM. *P <0.05, **P <0.01. Data in **E** were analyzed by two-tailed Student’s *t*-tests, and data in **F**, **H**, and **I** were analyzed by two-way ANOVA followed by Tukey’s *post hoc* test. GH: group housing; Re: resocialization; SI: social isolation.

To investigate the molecular mechanism by which Egr2 regulates myelinogenesis, we cultured primary OLs *in vitro*, transfected them with an Egr2-interfering plasmid post-differentiation, and collected the cells 48 h later for RNA sequencing (GSE272732) (Fig. 4A, B). GO enrichment analysis indicated that extracellular matrix enrichment is the most prominent biological process (Fig. 4B). Combined with PPI analysis, several genes directly related to Egr2, such as *Pdgfra*, *Serpinb5*, *Smad3*, *Tgfb2*, *Tgfb1*, *TNF*, *Tnfrsf1a*, *Gfap*, *Il6*, *Mmp3*, and *Nfkb2,* were screened (Fig. 4C, D). We further verified the aforementioned gene transcription levels using qPCR in primary OLs transfected with an Egr2-interfering plasmid, and found that the transcription levels of *Pdgfra*, *Tgfb2*, *Gfap*, and *Nfkb2* in the siEgr2 group were significantly increased (Fig. 4E). Next, we examined the transcription levels of the aforementioned four modified genes in the mPFC of the two resocialization modes. We discovered that, in comparison to the GH group, the SI group exhibited elevated *Pdgfra* gene levels in the mPFC, which were further increased in the Re-SI group but not in the Re-GH group (Fig. 4F). Immunofluorescent results further substantiated that the expression levels of PDGFRα in the mPFC of SI mice were reversed by resocialization with GH mice (Fig. 4G, H). SI mice, co-raised with SI companions exhibiting specific overexpression of Egr2 in mPFC OLs, demonstrated an increased expression level of PDGFRα in the mPFC (Fig. S11A, B). In contrast, SI mice resocialized with GH mice, where Egr2 in mPFC OLs was specifically interfered with, did not show an increase in PDGFRα expression in the mPFC (Fig. S11C, D). Collectively, these results suggest that the improved mPFC myelination in SI mice after resocialization with GH companions may be related to the down-regulation of PDGFRα.

**Fig. 5:**
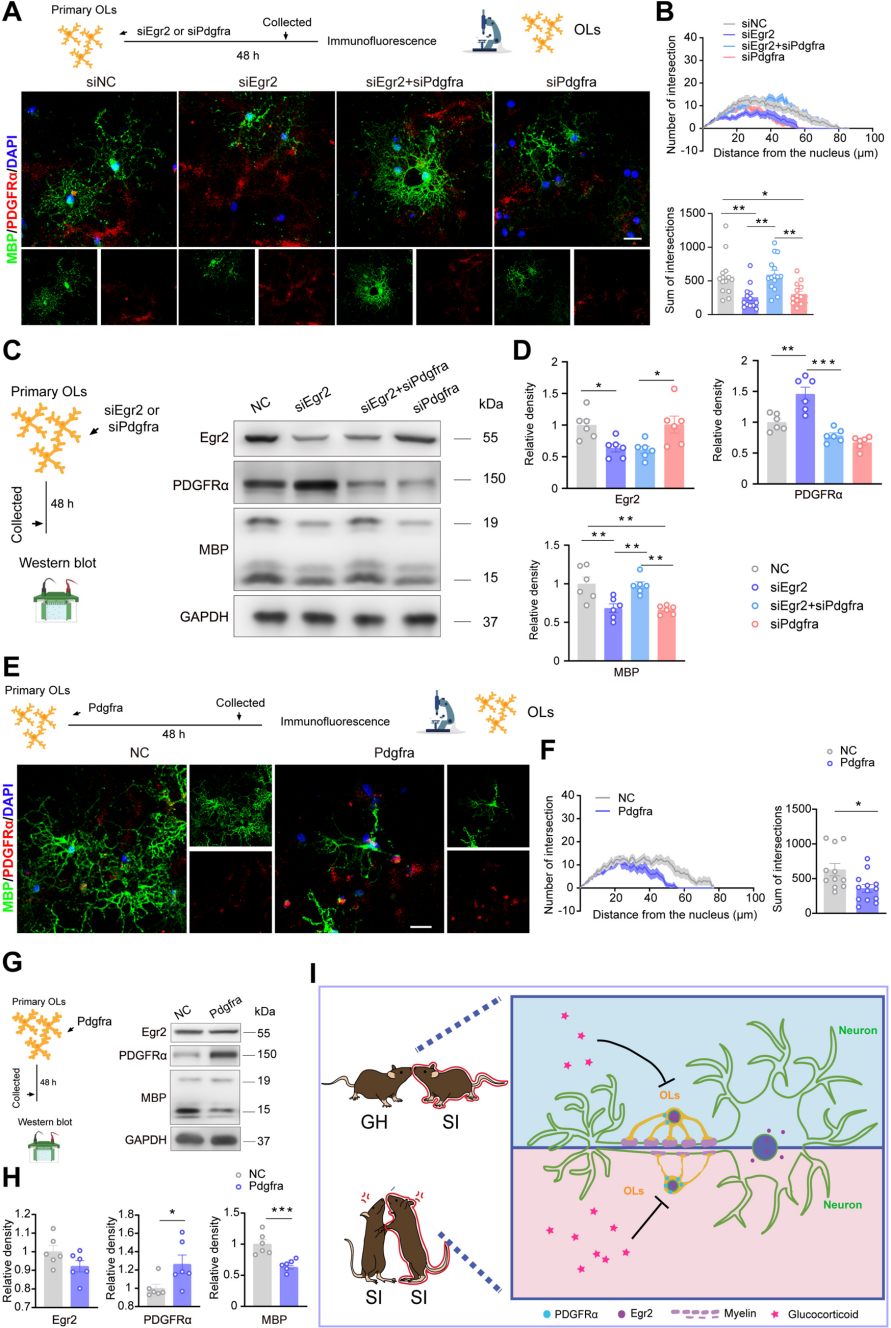
Egr2 inhibited PDGFRα expression in primary OLs. **A**, **B** Representative immunofluorescence images and Sholl analysis showing the differentiation status of primary OLs when treated with siEgr2 or siPdgfrα, including branch length and sum of intersections of OLs (14 cells for siNC; 13 cells for siEgr2; 14 cells for siPDGFRα; 15 cells for siEgr2+siPdgfrα) (*n* = 6). Scale bar, 50 µm. **C**, **D** Representative bands and the corresponding graphs showing Egr2, PDGFRα, and MBP (both molecular weights) protein expression in the OLs with or without siEgr2 and/or siPdgfrα (*n* = 6). **E**, **F** Representative immunofluorescence images and Sholl analysis identify that *Pdgfrα* (red) overexpression reduced branch length and the sum of intersections of primary OLs (green) (11 cells for siNC; 13 cells for PDGFRα) (*n* = 6). Scale bar, 50 µm. **G**, **H** Representative bands and corresponding graphs showing Egr2, PDGFRα, and MBP (both molecular weights) protein expression in the OLs with/or without the *Pdgfrα gene* (*n* = 6). **I** Resocialization with GH mice for 4 weeks can improve the loss of social behavior and myelin damage caused by isolation, and its mechanism may be that Egr2 negatively regulates the expression of PDGFRα in OLs, thereby promoting the differentiation of OLs and myelination. Data are presented as the mean ± SEM. *P <0.05, **P <0.01, ***P <0.001. Data in **B** and **D** were analyzed by two-way ANOVA followed by Tukey’s *post hoc* test, and data in **F** and **H** were analyzed by two-tailed Student’s *t* tests. GH: group housing; Re: resocialization; SI: social isolation.

To confirm the regulatory role of Egr2 on the maturation of OLs *via* PDGFRα, a dual luciferase reporter gene assay was performed. The results showed that Egr2 could directly bind to PDGFRα (Fig. 4I). Furthermore, quantitative analysis of ChIP indicated that Egr2 could precipitate within the *Pdgfrα* locus, binding at sites B1, B2, and B3 (Fig. 4J, K). Subsequently, we transfected interfering plasmids of Egr2 and/or PDGFRα into primary OL cultures. We observed that OLs in the siEgr2 group exhibited fewer and shorter branches. However, simultaneously interfering with the expression levels of PDGFRα in the primary OLs rescued the decline in branch complexity caused by siEgr2 (Fig. 5A, B). Western blot results consistently indicated that in the siEgr2 group, the expression levels of PDGFRα were significantly increased, whereas the expression levels of MBP were significantly decreased. Both effects were partially reversed by the transfection of siPdgfrα (Fig. 5C, D). The transcription of the overexpressed plasmid of PDGFRα led to a reduction in the branch length and total number of branches of primary OLs, as well as a decrease in MBP expression (Fig. 5E–H). In summary, these findings suggest that Egr2 promotes myelination by transcriptionally suppressing the expression of PDGFRα in OLs of SI mice, thereby contributing to the enhancement of their social abilities.

## Discussion

Social creatures, including humans, rely on their peers for the establishment, maintenance, and enhancement of their social behavior capabilities [29]. For instance, a recent study indicated that the academic performance of college students is highly correlated with the intimacy and diversity of their roommates [30]. Thus, various social interaction partners have distinct impacts on individuals, although the underlying mechanisms are not fully understood. In this study, we demonstrated that SI mice experienced reduced mPFC myelin and synaptic damage and improved social behavior after being co-housed with GH mice, whereas resocialization with other SI mice only mitigated synaptic damage. Targeting the expression of Egr2 specifically in mPFC OLs of SI mice and their resocialized counterparts confirmed its crucial role in resocialization, enhancing myelination and social behavior capabilities. Further results indicated that isolation resulted in elevated Cort levels, and during the resocialization phase, SI mice’s companions who received intraperitoneal injections of Cort exhibited inhibited OL Egr2 levels in the mPFC of SI mice, thus failing to repair their PFC hypomyelination and social ability defects. In contrast, SI mice’s companions treated with Mety during resocialization promoted OL Egr2 levels in SI mice, thereby enhancing mPFC myelination and alleviating social cooperation impairments. *In vivo* and *in vitro* experiments further demonstrated that Egr2 facilitated myelin formation by inhibiting PDGFRα, thereby promoting OL differentiation and maturation. Collectively, OL Egr2 is essential for resocialization to rescue social ability defects caused by early SI (Fig. 5I).

Childhood is a critical period for the establishment of capacity and style, and it is susceptible to external influences. Epidemiological investigations indicate that socially isolated adolescents are more likely to develop physical developmental disorders and mental health problems in adulthood [31]. Therefore, timely and effective interventions for adolescent behavioral abnormalities are particularly crucial. Resocialization has been shown to benefit children’s development with early SI or adverse social experiences [7]. Previous studies have indicated that appropriate psychosocial interventions, such as resocialization, can reverse the thinning of myelin and the associated neuronal activity deficits in the mPFC caused by juvenile social isolation [13]. In our study, we induced adolescent SI by raising mice in isolation for four weeks post-weaning. We then assessed their social abilities using cooperative behavior, three-chamber experiments, and prosocial behavior. Our findings revealed that mice raised in isolation exhibited significantly impaired social abilities. However, resocialization with normal GH mice enhanced the social abilities of SI mice, whereas resocialization with other SI mice did not result in significant improvements. These results suggest that the type of companions plays a crucial role in correcting social behavioral deficits.

It is important to note that cooperative behavior, as a complex and advanced social behavior, appears to depend on the dual integrity of myelin and synapses [11]. This study further revealed that following 3 weeks of isolation post-weaning, resocialization with GH mice enhanced myelination and synaptic integrity in the mPFC, whereas resocialization with SI companions only restored synaptic damage. Previous studies have shown that synaptic development and maturation in the central nervous system (CNS) are completed by 3 weeks of age in mice, while myelination occurs later and continues until the end of adolescence [32]. Short-term isolation during early adolescence, for instance, from postnatal day 30 to 35, may result in synaptic loss in the mPFC [33, 34]. However, prolonged isolation throughout adolescence can halt typical synaptic pruning, leading to an overabundance of spines in adulthood [35]. It has been reported that synaptic dysfunction induced by SI can be restored through increased EphB2 expression in the hippocampal CA1 region, a process facilitated by resocialization [36]. Additionally, long-term social isolation has persistently negative effects on myelination in the mPFC [12, 24]. The present results showed that resocialization with either GH or SI companions rescued impaired mPFC synaptic integrity. The different mechanisms of resocialization on the myelin sheath and synaptic repair deserve further investigation. Previous studies have indicated that the social behavior of isolated female mice is not as significantly impaired as that of male mice, which may be attributed to the protective effects of estrogen against stress [37, 38]. During chronic SI, the PFC-BLA circuit mediates an increase in aggression in male mice, whereas the PFC-VTA circuit is implicated in a reduced increase in discharge rates during sociability tests in female mice [39, 40]. Future research could further explore the impact of gender differences on social cooperation between males and females.

The role of Egr2 in myelination is well established in the peripheral nervous system [41]. Egr2 promotes the transition of Schwann cells to the differentiated myelination stage [42]. It has also been reported that Egr2 exists in the CNS [43]. In particular, Marques *et al*. identified a specific population of Egr2-expressing OLs in the CA1 hippocampus using single-cell sequencing [43]. Egr2 is expressed in various brain regions, including the hippocampus, olfactory bulb, neocortex, amygdala, striatum, diencephalic structures, cerebellum, and brainstem [44, 45]. It is worth exploring how Egr2 also regulates the diverse behavioral effects of SI in various brain regions. Our recent studies have found that social cooperation can only be improved by Egr2 overexpression in the mPFC of SI mice driven by a broad-spectrum promoter, while specific overexpression of Egr2 in neurons or OLs is not sufficient to improve the social cooperation of these SI mice [11]. In this study, SI mice resocialized with SI companions that overexpress Egr2 on mPFC OLs showed improvements in damaged myelin sheaths and synapse integrity, as well as social cooperation disorders. Overexpression of Egr2 in mPFC OLs of SI mice can restore social interaction performance in the three-chamber test [11]. The current study further underscores the importance of OL Egr2 in the mPFC for establishing social ability during resocialization. However, considering the sample size and workload of the experimental mice and avoiding the repeated reporting, we did not assess the behavioral and pathological changes in these virus-treated mice. During resocialization, neurotrophic factors such as microglia-derived neuregulin-1 [46] and brain-derived neurotrophic factor (BDNF) [47] have been reported to influence neuronal function and behavior. Neuregulin-1 and BDNF can prompt OLs to shift towards NMDA receptor-dependent myelin formation, thereby participating in myelin regeneration following white matter injury [48]. The neuroregulin-1-ErbB signal transduction pathway is linked to the adverse effects of early social experiences, which is also crucial for OL maturation [49, 50].

Our previous study reported that Cort treatment suppressed Egr2 expression levels in OLs [11]. Indeed, isolation during adolescence is associated with heightened reactivity of the HPA axis in the PFC [51]. SI triggers the activation of the HPA axis, resulting in elevated plasma Cort levels [52]. However, the stress response of the HPA axis varies depending on the duration and patterns of SI stress. Maternal separation in newborn mice has been shown to reduce Cort levels [53], whereas isolation rearing during adolescence and adulthood has been associated with increased GC levels [54, 55]. The current study found that the increased serum Cort levels in mice isolated for 3 weeks post-weaning were reversed by resocialization with GH mice, leading to enhancements in social ability in SI mice. Peripheral Cort supplementation exacerbated the resocialization abilities of "donor" mice, negating their reparative effects on mPFC hypomyelination and deficits in social behavior of SI mice.

Studies have demonstrated that although acute increases in GCs can promote the differentiation of neuronal stem cells and primary oligodendrocyte precursor cells (OPCs) by activating glucocorticoid receptors, thereby enhancing myelination, prolonged exposure to Cort has a detrimental effect on myelin [56]. In individuals with major depressive disorder, elevated Cort levels are linked to decreased white matter integrity in the frontal-cortical and frontal-limbic systems [57], whereas the expression of myelin-related genes such as *Mag* and *Cnp* is notably increased in brains with lower Cort levels [58]. Cort also indirectly impedes myelination by opposing the effects of pro-oligodendrogenesis hormones [56]. In line with these findings, SI mice with normalized elevated Cort levels exhibited enhancements in mPFC myelination and OL differentiation in this study.

Previous studies have shown that glucocorticoids regulate neuroinflammation and oligodendrocyte function *via* their receptors (GR and MR) [59, 60]. It has also been demonstrated that glucocorticoids can affect myelin formation either directly or indirectly [61, 62]. The use of glucocorticoid synthesis inhibitors (such as metyrapone) or receptor antagonists (such as RU486) may effectively mitigate the adverse effects of social isolation on myelin integrity. Indeed, RU486 has been reported to alleviate glucocorticoid-related pathological features in various neurological disease models [63], indicating its potential therapeutic significance. Nevertheless, the exact effectiveness and safety of these medications still need thorough exploration in the future.

The development and maturation of OLs, as well as the differentiation of OPCs, are mediated by numerous intrinsic and extrinsic factors throughout life [64]. *Pdgfrα*, a gene encoding a cell surface tyrosine kinase receptor, is one of the intrinsic factors that specify the OPC lineage [65] and is crucial for the growth and maintenance of OPCs in the CNS [66]. Previous studies have suggested that mice with *Pdgfrα* knockout exhibited developmental defects in OLs, manifesting as severe myelin dysplasia [67]. The overexpression of human PDGFRα in mice also disrupted OL development, leading to hypomyelination [66]. Consequently, the role of PDGFRα in myelin formation appears to be bidirectional. Appropriately reducing PDGFRα signaling appears to be beneficial for OL maturation and myelin regeneration [68]. The present results further discovered that Egr2 in the mPFC OLs directly inhibited the expression of PDGFRα in OLs, which promoted myelin formation to a certain extent. This finding provides new insights into the regulatory mechanisms of myelin formation and social behavior.

In summary, this study demonstrated that reintroducing SI adolescent mice into a social environment with GH mice improved their myelination deficits and social abilities. In reality, the impact and factors of early SI in human society are far more extensive. Notably, nearly three-quarters of adult mental disorders originate from childhood [69]. The current findings provide a theoretical basis for early intervention in adolescents with SI. Appropriate social therapeutic approaches might rescue early negative life experiences related to hypomyelination and social behavior defects, which can be applied to clinical research for orphans or left-behind children.

## Supplementary Information

Below is the link to the electronic supplementary material.

Supplementary file1 (PDF 5355 KB)
