# Supplementary material for: Oligodendrocyte Egr2 Mediates the Beneficial Effect of Resocialization on the Social Ability of Socially Isolated Mice
Source: Neurosci Bull. 2025 Nov 6;42(6):1261–78. doi: 10.1007/s12264-025-01534-w (PMC13221552; doi:10.1007/s12264-025-01534-w)
Supplement: Supplementary file 1 — Supplementary file1 (PDF 5355 KB) [file 12264_2025_1534_MOESM1_ESM.pdf]

Supplementary Materials

Supplementary Figures

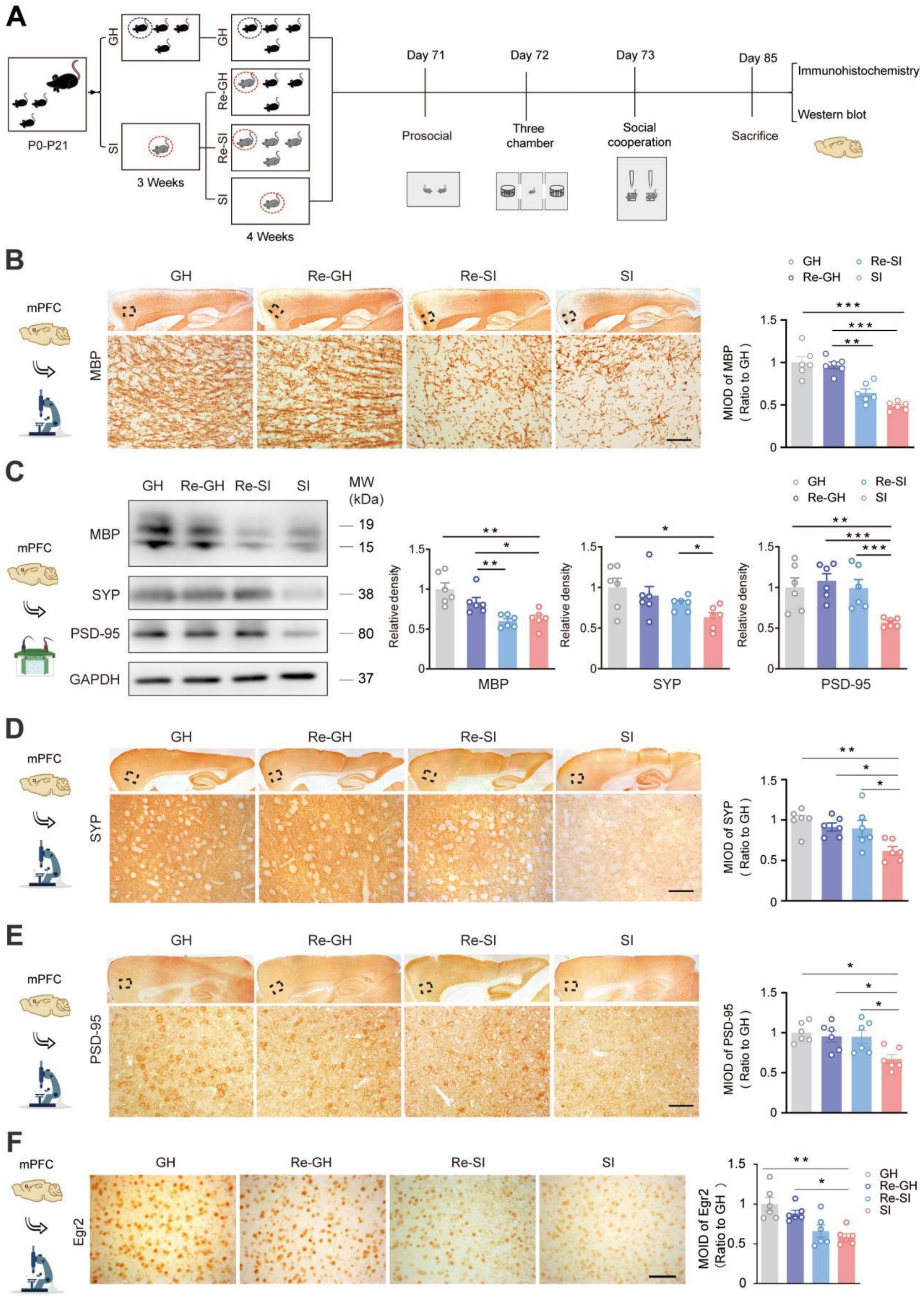

Fig. S1 Resocialization with GH mice, but not with SI mice, improved mPFC myelin protein loss in SI mice. **A** Schematic diagram showing the timeline of the experimental design for two resocialization modes of SI mice. **B** Representative immunohistochemical images and corresponding graphs showing MBP expression in the mPFC of mice (*n* = 6). Scale bar, 50 μm. **C** Representative bands and corresponding graphs showing MBP (both molecular weights), SYP, and PSD-95 protein expression in the mPFC of mice (*n* = 6). **D–F** Representative immunohistochemical images and corresponding graphs showing SYP (**D**), PSD-95 (**E**), and Egr2 (**F**) expression in the mPFC of mice (*n* = 6). Scale bars, 50 μm. Data are presented as the mean ± SEM. **P* <0.05, ***P* <0.01, ****P* <0.001. Data were analyzed using two-way ANOVA followed by Tukey's *post hoc* test. GH: group housing; Re: resocialization; SI: social isolation; mPFC: medial prefrontal cortex.

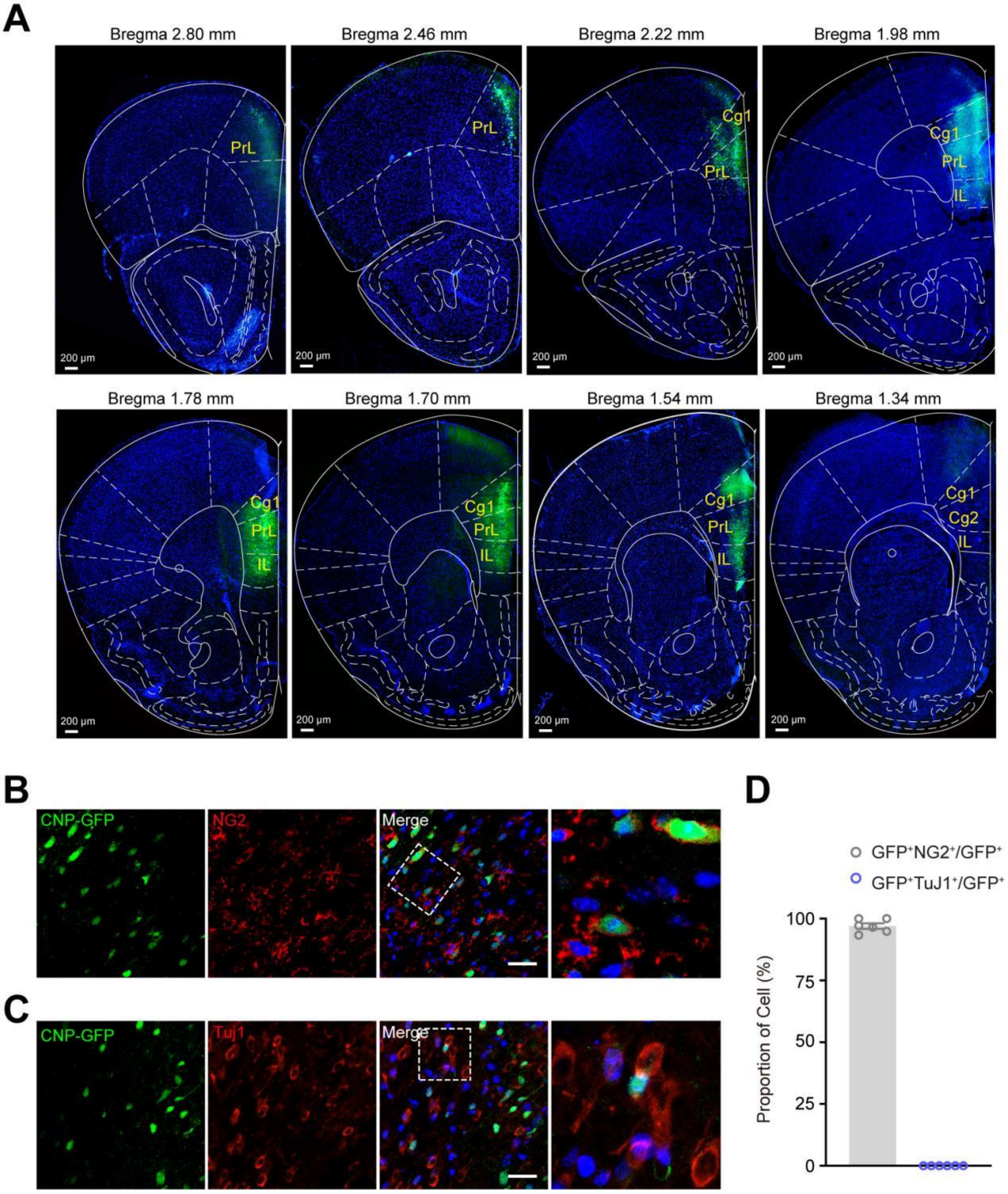

Fig. S2 The verification of the CNP promoter for oligodendrocyte-specific manipulation. **A** The extent of CNP-GFP-positive virus spreading from the anterior to the posterior prefrontal cortex. Scale bars, 200 μm. **B** Representative image showing the $NG2^+$ (red) cells were mostly co-localized with GFP^+ cells. Scale bar, 50 μm. **C** Representative images showing the $Tuj1^+$ (red) cells were not co-localized with GFP^+ cells. Scale bar, 50 μm. **D** The statistical graph shows the percentage of $NG2^+GFP^+$ cells

and Tuj1$^+$GFP$^+$ cells after injection of CNP promoter virus into the mPFC (n = 6). Data are presented as the mean ± SEM.

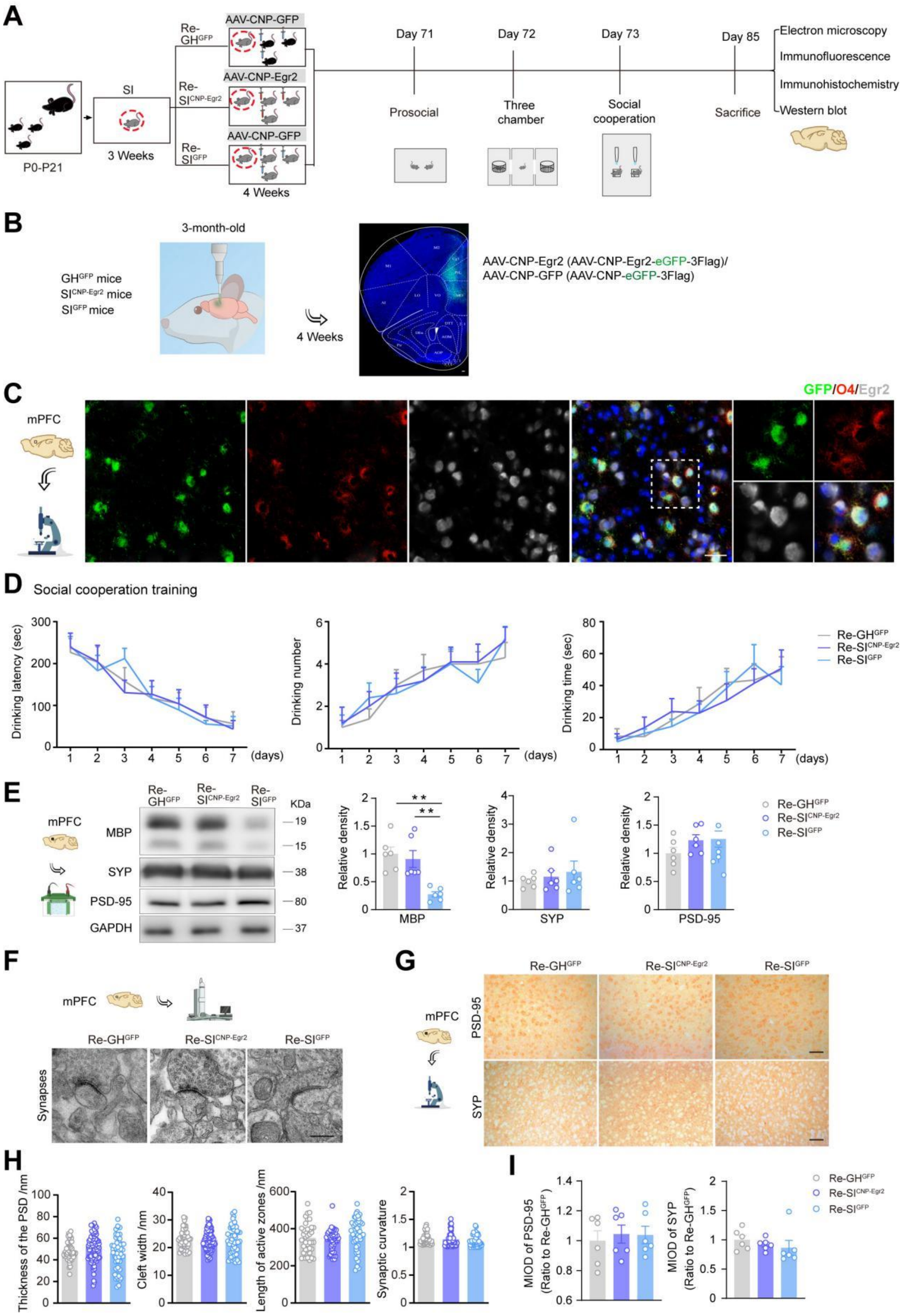

Fig. S3 Resocialization of SI mice with overexpression of Egr2 in mPFC OLs improved synaptic damage in SI mice. **A** Schematic diagram showing the timeline of the experimental design for SI mice resocialization with SI mice that overexpressed Egr2 in mPFC OLs. **B, C** Representative images showing the effective validation of AAV virus injection. Scale bars, 50 μm. **D** Bar graphs showing the drinking latency, drinking number, and drinking time of mice in the training period (n = 10). **E** Representative bands and corresponding graphs showing MBP (both molecular weights), SYP, and PSD-95 protein expression in the mPFC of mice (n = 6). **F** Representative EM images showing synaptic morphology in the mPFC of mice. Scale bar, 500 nm. **G** Representative immunohistochemical images showing PSD-95 and SYP expression in the mPFC of mice (n = 6). Scale bars, 50 μm. **H, I** Bar graphs showing quantification of PSD, synaptic cleft width, length of the active zones, and synaptic curvature (Re-GH^{GFP}: 55 synapses; Re-SI$^{CNP\text{-}Egr2}$: 54 synapses; Re-SI^{GFP}: 56 synapses) (**H**), PSD-95 and SYP expression (**I**) in the mPFC of mice (n = 6). Data are presented as the mean ± SEM. *P <0.05, **P <0.01. Data in **D** were analyzed by repeated-measures ANOVA with *post hoc* Student-Newman-Keuls test, and others were analyzed by one-way ANOVA followed by Tukey's *post hoc* test. GH: group housing; Re: resocialization; SI: social isolation; mPFC: medial prefrontal cortex.

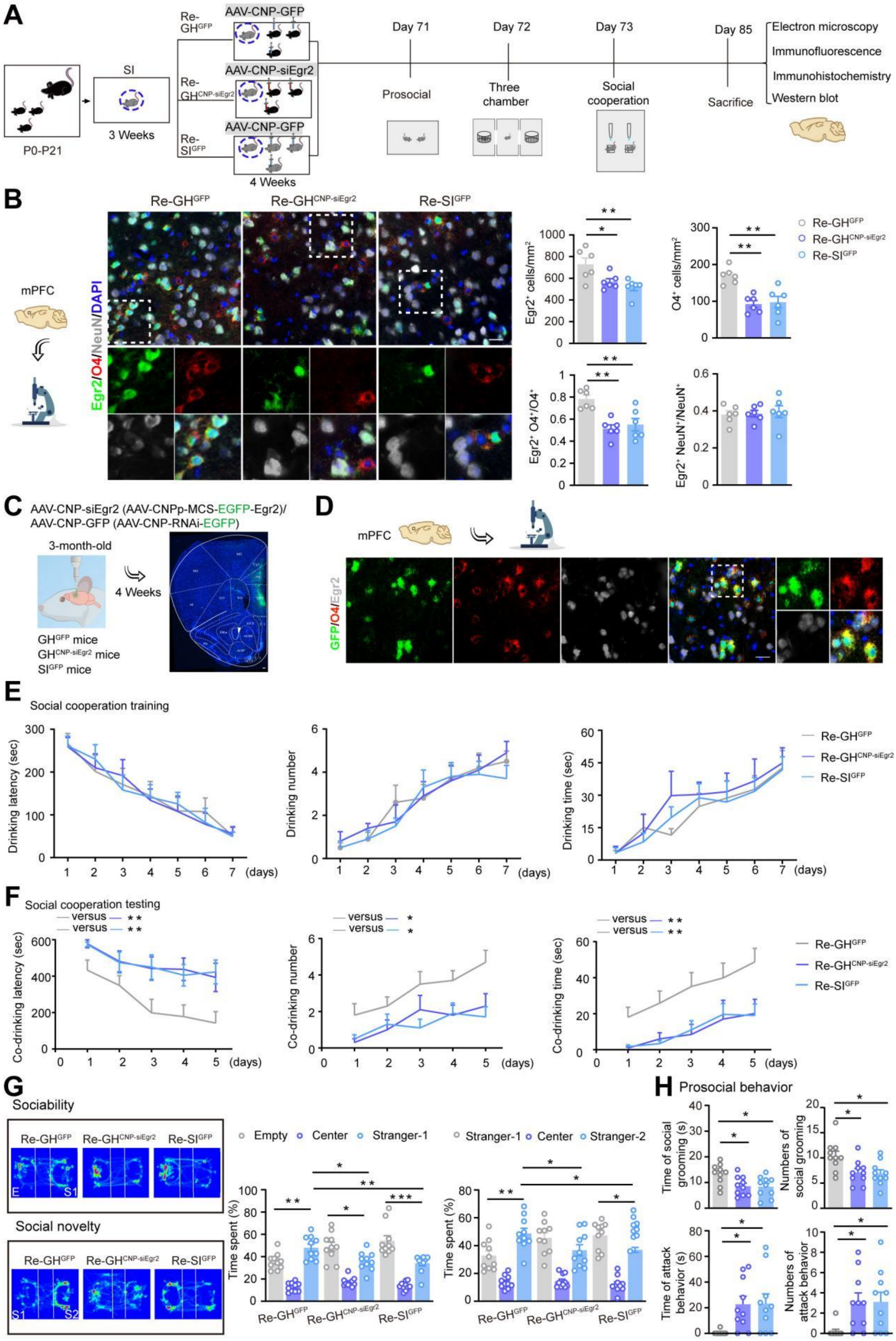

A
P0-P21
SI
3 Weeks
Re-GHGFP
Re-GHCNP-siEgr2
Re-SIGFP
AAV-CNP-GFP
AAV-CNP-siEgr2
AAV-CNP-GFP
4 Weeks
Day 71
Prosocial
Day 72
Three chamber
Day 73
Social cooperation
Day 85
Sacrifice
Electron microscopy
Immunofluorescence
Immunohistochemistry
Western blot
B
mPFC
Egr2/O4/NeuN/DAPI
Egr2+ cells/mm2
O4+ cells/mm2
Egr2+ O4+/O4+
Egr2+ NeuN+/NeuN+
C
AAV-CNP-siEgr2 (AAV-CNPp-MCS-EGFP-Egr2)/
AAV-CNP-GFP (AAV-CNP-RNAi-EGFP)
3-month-old
4 Weeks
GHGFP mice
GHCNP-siEgr2 mice
SIGFP mice
D
mPFC
GFP/O4/Egr2
E
Social cooperation training
Drinking latency (sec)
Drinking number
Drinking time (sec)
(days)
F
Social cooperation testing
versus
Co-drinking latency (sec)
Co-drinking number
Co-drinking time (sec)
G
Sociability
Social novelty
Empty
Center
Stranger-1
Stranger-2
Time spent (%)
H
Prosocial behavior
Time of social grooming (s)
Numbers of social grooming
Time of attack behavior (s)
Numbers of attack behavior

Fig. S4 The social ability of SI mice was not enhanced through resocialization with GH mice that had Egr2 knocked down in mPFC OLs. **A** Schematic diagram showing the timeline of the experimental design for SI mice resocialization with GH mice that knock down Egr2 in mPFC OLs. **B** Representative immunofluorescence images and the corresponding graph showing $Egr2^{+}$ (green), $O4^{+}$ (red), $O4^{+}$ $Egr2^{+}/O4^{+}$, and $NeuN^{+}$ (gray) $Egr2^{+}/NeuN^{+}$ expression in the mPFC of mice (n = 6). Scale bar, 50 µm. **C, D** Representative images showing the effective validation of AAV virus injection. Scale bars, 50 µm. **E** Bar graphs showing the drinking latency, drinking number, and drinking time of mice in the training period (n = 10). **F** Bar graphs showing the co-drinking latency, co-drinking number, and co-drinking time of mice in the social cooperation test (n = 10). **G** Heat map and bar graphs showing the sociability and social novelty of mice evaluated by the three-chamber test (n = 10). **H** Chart showing the time and number of social grooming and attacks during the prosocial behavior in mice (n = 10). Data are presented as the mean ± SEM. *P <0.05, **P <0.01, ***P <0.001. Data in **E** and **F** were analyzed by repeated-measures ANOVA with *post hoc* Student–Newman–Keuls test, data in **G** were analyzed by two-way ANOVA followed by Tukey's *post hoc* test, and others were analyzed by one-way ANOVA followed by Tukey's *post hoc* test. GH: group housing; Re: resocialization; SI: social isolation; mPFC: medial prefrontal cortex.

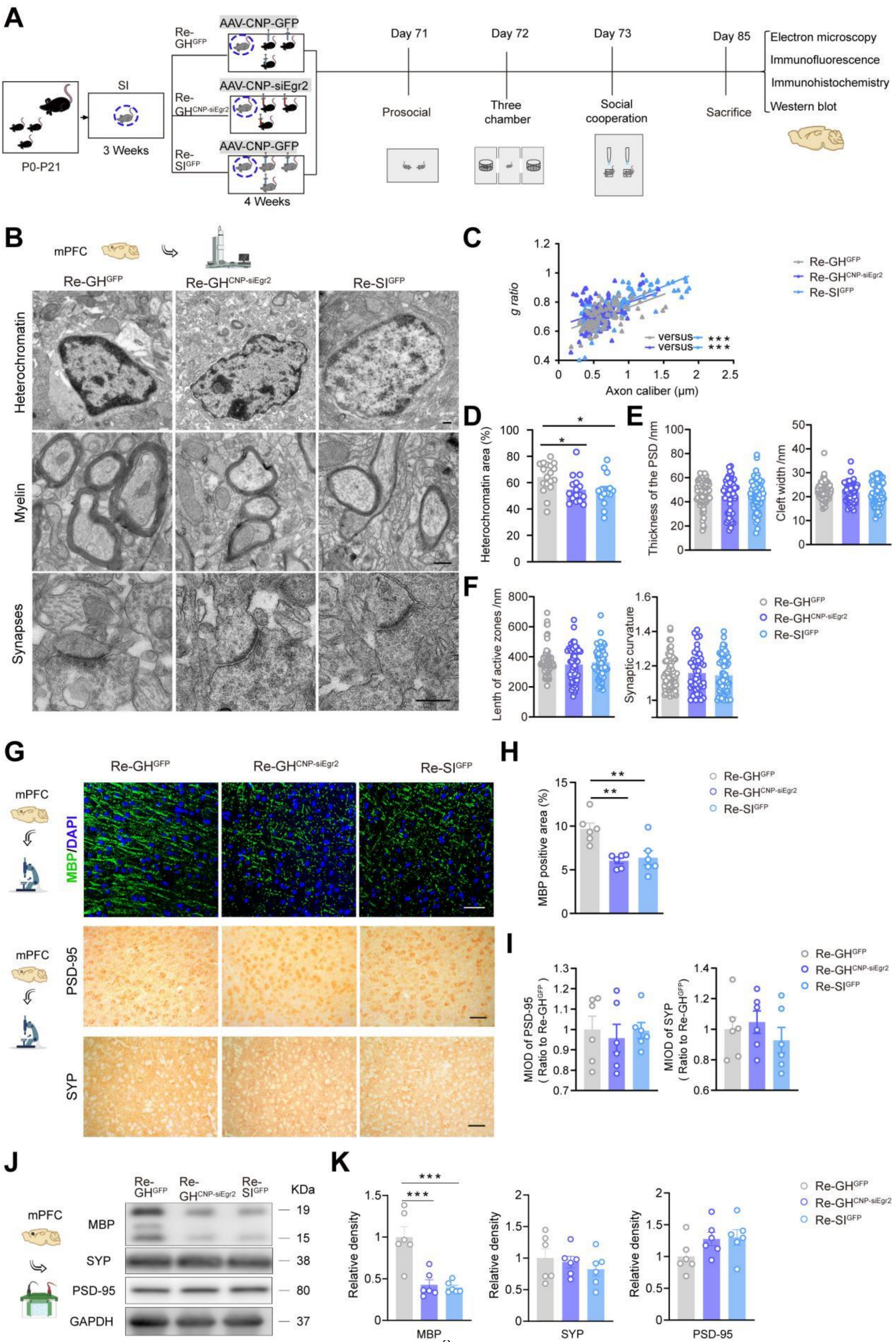
A
P0-P21
SI
3 Weeks
Re-GHGFP
Re-GHCNP-siEgr2
Re-SIGFP
AAV-CNP-GFP
AAV-CNP-siEgr2
AAV-CNP-GFP
4 Weeks
Day 71
Day 72
Day 73
Day 85
Prosocial
Three chamber
Social cooperation
Sacrifice
Electron microscopy
Immunofluorescence
Immunohistochemistry
Western blot
B
mPFC
Re-GHGFP
Re-GHCNP-siEgr2
Re-SIGFP
Heterochromatin
Myelin
Synapses
C
g ratio
Axon caliber (μm)
versus ***
versus ***
D
Heterochromatin area (%)
E
Thickness of the PSD /nm
Cleft width /nm
F
Lenth of active zones /nm
Synaptic curvature
G
MBP/DAPI
PSD-95
SYP
H
MBP positive area (%)
I
MIOD of PSD-95 (Ratio to Re-GHGFP)
MIOD of SYP (Ratio to Re-GHGFP)
J
MBP
SYP
PSD-95
GAPDH
KDa
19
15
38
80
37
K
Relative density
MBP
SYP
PSD-95

Fig. S5 Resocialization of GH mice with Egr2 knockdown in mPFC OLs improved synapses rather than myelin in SI mice. **A** Schematic diagram showing the timeline of the experimental design for SI mice resocialization with GH mice that knock down Egr2 in mPFC OLs. **B** Representative EM images showing OLs nuclear heterochromatin, myelin, and synaptic morphology in the mPFC of mice. Scale bars, 500 nm. **C** Scatter plot of *g*-ratios with linear least squares fitting (Re-GH^{GFP}: 209 axons; Re-$GH^{CNP\text{-}siEgr2}$: 218 axons; Re-SI^{GFP}: 193 axons) (n = 6). **D** Graph showing the area percentage of OLs nuclear heterochromatin (Re-GH^{GFP}: 17 nuclei; Re-$GH^{CNP\text{-}siEgr2}$: 17 nuclei; Re-SI^{GFP}: 16 nuclei) (n = 6). **E, F** Bar graphs showing quantification of PSD, synaptic cleft width, length of the active zones, and synaptic curvature (Re-GH^{GFP}: 53 synapses; Re-$GH^{CNP\text{-}siEgr2}$: 58 synapses; Re-SI^{GFP}: 54 synapses). **G–I** Representative immunohistochemical images showing MBP (green), PSD-95, and SYP expression in the mPFC of mice (n = 6). **J, K** Representative bands and corresponding graphs showing MBP (both molecular weights), SYP, and PSD-95 protein expression in the mPFC of mice (n = 6). Data are presented as the mean ± SEM. *P <0.05, **P <0.01, ***P <0.001. Data in **C** were analyzed by repeated-measures ANOVA with *post hoc* Student–Newman–Keuls test, and others were analyzed by one-way ANOVA followed by Tukey's *post hoc* test. GH: group housing; Re: resocialization; SI: social isolation; mPFC: medial prefrontal cortex.

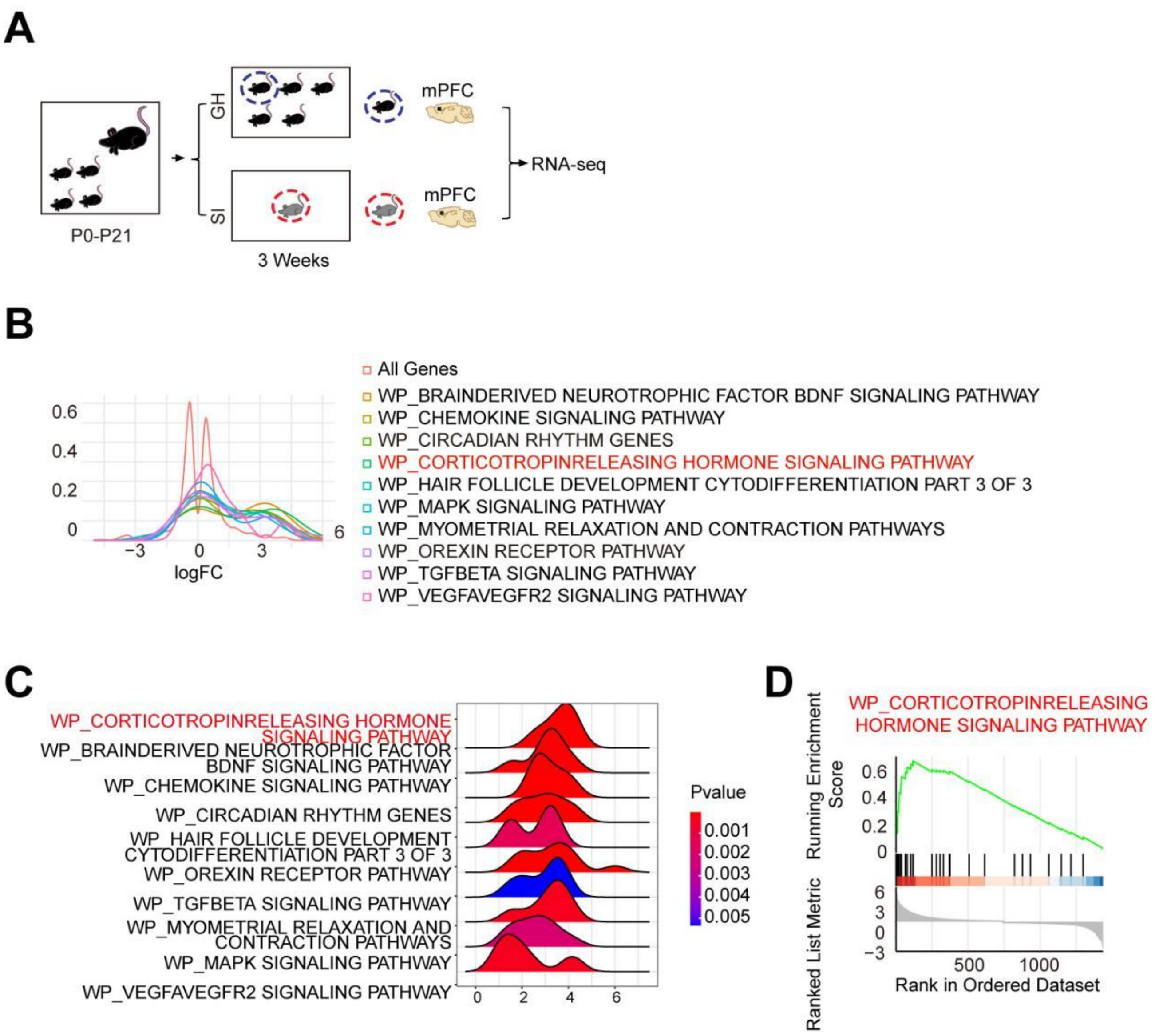

Fig. S6 RNA-seq analysis of the mPFC in SI mice compared to GH mice. **A** Schematic diagram showing the timeline of the experimental design for RNA sequencing of the mPFC in SI mice and GH mice. **B–D** GSEA analysis showing the enrichment in corticotropin-releasing hormone signaling pathway. GH: group housing; SI: social isolation; mPFC: medial prefrontal cortex.

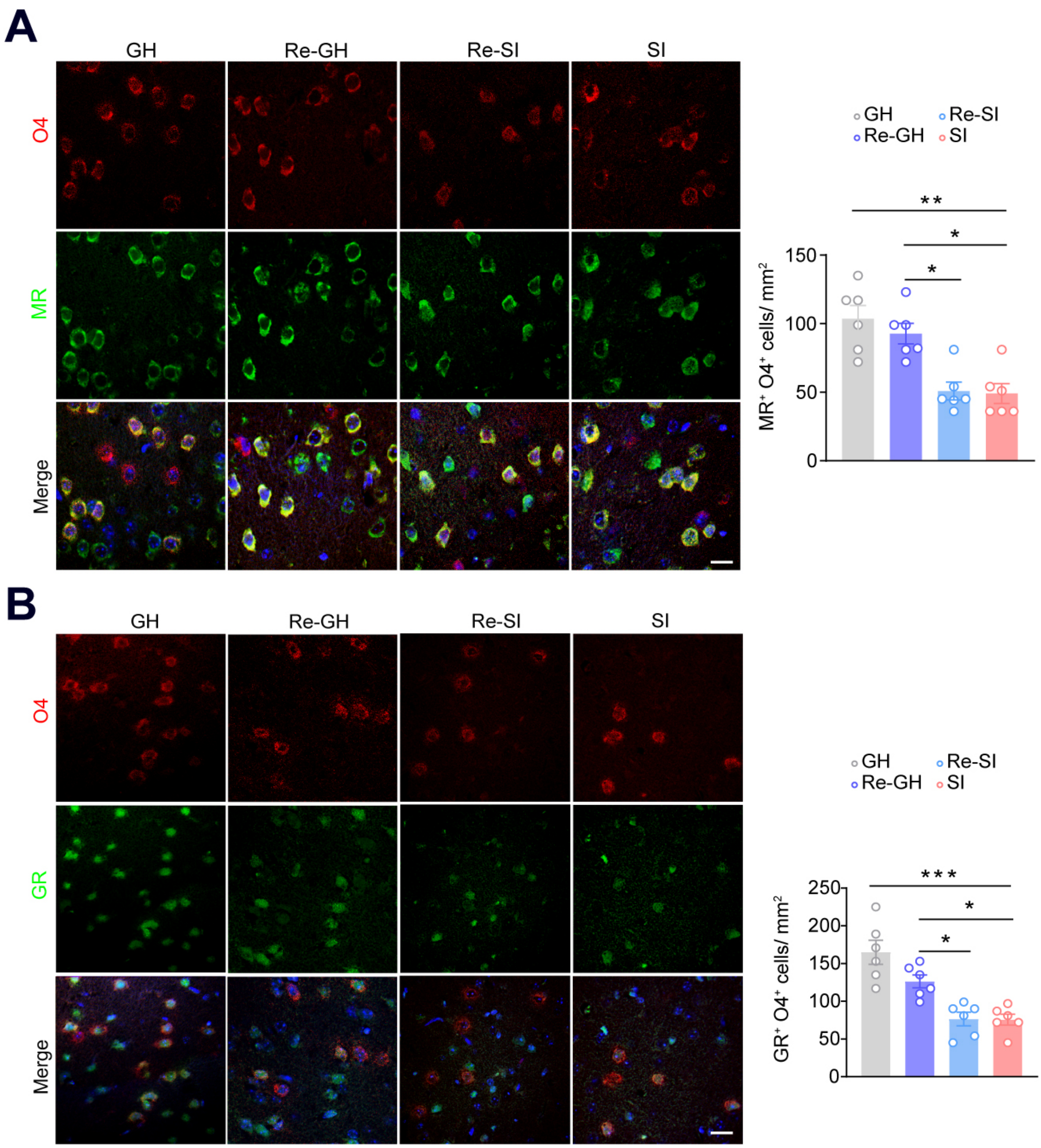

Fig. S7 Resocialization with GH mice, but not with SI mice, improved mPFC GR and MR expression in oligodendrocytes. **A** Representative immunofluorescence images and the corresponding graph showing MR$^+$ (green) O4$^+$ (red) expression in the mPFC of SI mice resocialized with GH mice or SI mice (n = 6). Scale bar, 50 μm. **B** Representative immunofluorescence images and the corresponding graph showing GR$^+$ (green) O4$^+$ (red) expression in the mPFC of SI mice resocialized with GH mice

or SI mice (n = 6). Scale bar, 50 μm. Data are presented as the mean ± SEM. *P <0.05, **P <0.01, ***P <0.001. Data were analyzed using two-way ANOVA followed by Tukey's *post hoc* test. GH: group housing; Re: resocialization; SI: social isolation.

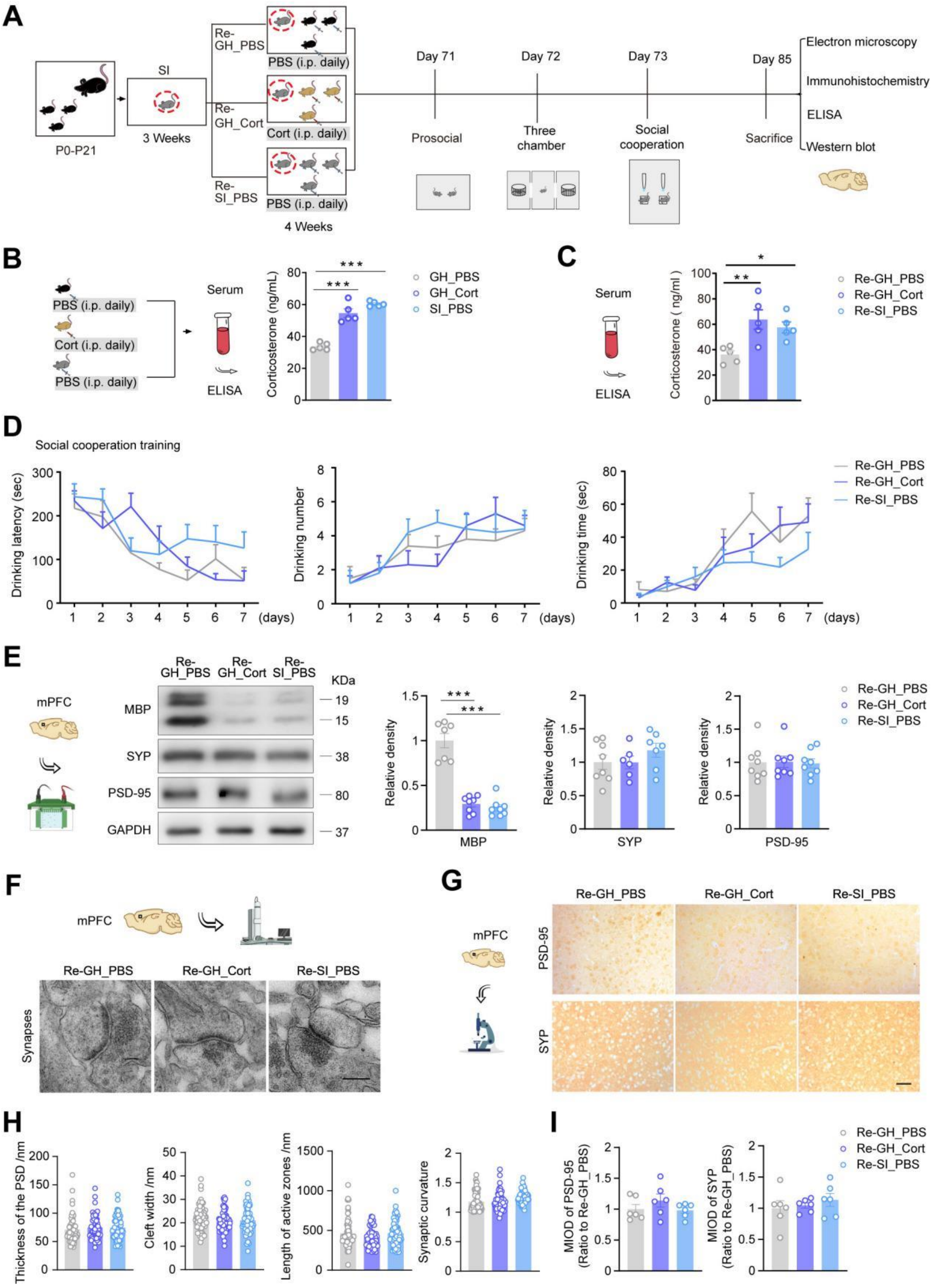

Fig. S8 Resocialization with GH mice that were intraperitoneally injected with Cort had no effect on synapses in the mPFC of SI mice. **A** Schematic diagram showing the timeline of the experimental design for SI mice housing with GH mice intraperitoneal injected with Cort. **B, C** Cort expression levels in serum of SI mice's companions treated by Cort (**B**), and SI mice (**C**), detected by ELISA (*n* = 6). **D** Bar graphs showing the drinking latency, drinking number, and drinking time of mice in the training period (*n* = 10). **E** Representative bands and corresponding graphs showing MBP (both molecular weights), SYP, and PSD-95 protein expression in the mPFC of mice (*n* = 6). **F** Representative EM images showing synaptic morphology in the mPFC of mice. Scale bar, 500 nm. **G** Representative immunohistochemical images showing PSD-95 and SYP expression in the mPFC of mice (*n* = 6). Scale bar, 50 μm. **H, I** Bar graphs showing quantification of PSD, synaptic cleft width, length of the active zones, and synaptic curvature (Re-GH_PBS: 62 synapses; Re-GH_Cort: 59 synapses; Re-SI_PBS: 60 synapses) (*n* = 6) (**H**), PSD-95 and SYP expression (**I**) in the mPFC of mice (*n* = 6). Data are presented as the mean ± SEM. *P <0.05, **P <0.01, ***P <0.001. Data in **D** were analyzed by repeated-measures ANOVA with *post hoc* Student-Newman-Keuls test, and others were analyzed by one-way ANOVA followed by Tukey's *post hoc* test. GH: group housing; Re: resocialization; SI: social isolation; mPFC: medial prefrontal cortex; Cort: corticosterone.

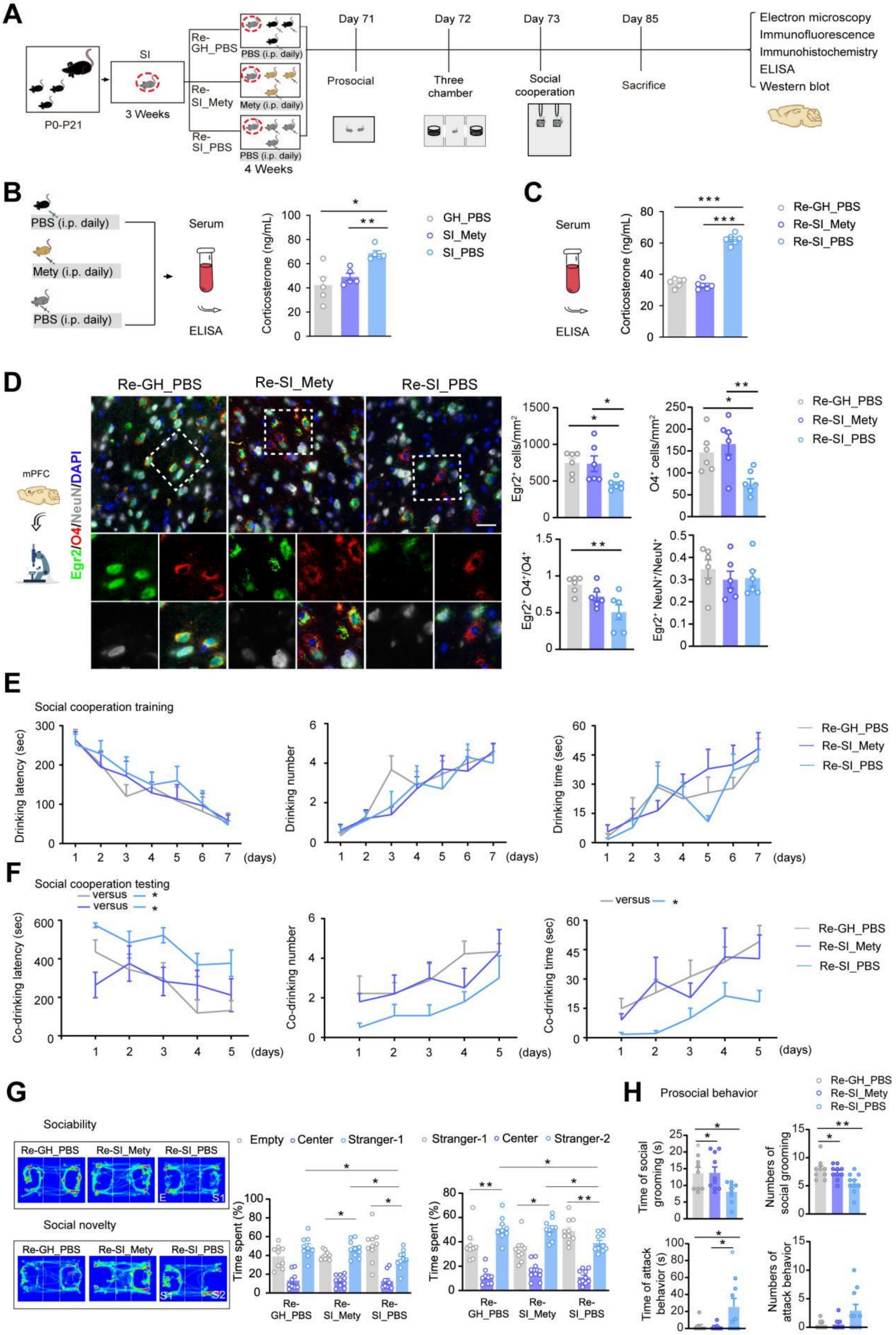
A
P0-P21
SI
3 Weeks
Re-GH_PBS
Re-SI_Mety
Re-SI_PBS
PBS (i.p. daily)
Mety (i.p. daily)
4 Weeks
Day 71
Prosocial
Day 72
Three chamber
Day 73
Social cooperation
Day 85
Sacrifice
Electron microscopy
Immunofluorescence
Immunohistochemistry
ELISA
Western blot
B
Serum
ELISA
Corticosterone (ng/mL)
GH_PBS
SI_Mety
SI_PBS
C
Re-GH_PBS
Re-SI_Mety
Re-SI_PBS
D
mPFC
Egr2/O4/NeuN/DAPI
Egr2⁺ cells/mm²
O4⁺ cells/mm²
Egr2⁺ O4⁺/O4⁺
Egr2⁺ NeuN⁺/NeuN⁺
E
Social cooperation training
Drinking latency (sec)
Drinking number
Drinking time (sec)
(days)
F
Social cooperation testing
versus
Co-drinking latency (sec)
Co-drinking number
Co-drinking time (sec)
G
Sociability
Social novelty
Empty
Center
Stranger-1
Stranger-2
Time spent (%)
H
Prosocial behavior
Time of social grooming (s)
Numbers of social grooming
Time of attack behavior (s)
Numbers of attack behavior

Fig. S9 SI companions treated with Mety exhibited improved social abilities in SI mice during resocialization. **A** The timeline of the experimental design. **B, C** ELISA results showing Cort levels in serum of SI mice's companions treated by Mety (**B**), and SI mice (**C**) (n = 5–6). **D** Representative immunofluorescence images and the corresponding graph showing $Egr2^+$ (green), $O4^+$ (red), $O4^+$ $Egr2^+/O4^+$, $NeuN^+$ (gray) $Egr2^+/NeuN^+$ expression in the mPFC of SI mice resocialized with GH mice with Cort treatment (n = 6). Scale bar, 50 μm. **E** Bar graphs showing the drinking latency, drinking number, and drinking time of mice in the training period (Re-GH_PBS: n = 9; Re-SI_Mety: n = 10; Re-SI_PBS: n = 10). **F** Bar graphs showing the co-drinking latency, co-drinking number, and co-drinking time of mice in the testing stage (Re-GH_PBS: n = 9; Re-SI_Mety: n = 10; Re-SI_PBS: n = 10). **G** Heat map and bar graphs showing the sociability and social novelty of mice evaluated by the three-chamber test (Re-GH_PBS: n = 9; Re-SI_Mety: n = 10; Re-SI_PBS: n = 10). **H** Chart showing the time and number of social grooming and attack during the prosocial behavior in mice (Re-GH_PBS: n = 9; Re-SI_Mety: n = 10; Re-SI_PBS: n = 10). Data are presented as the mean ± SEM. *P <0.05, **P <0.01, ***P <0.001. Data in **E, F** were analyzed by repeated-measures ANOVA with *post hoc* Student-Newman-Keuls test, data in **G** were analyzed by two-way ANOVA followed by Tukey's *post hoc* test, and in others were analyzed by one-way ANOVA followed by Tukey's *post hoc* test. GH: group housing; Re: resocialization; SI: social isolation; mPFC: medial prefrontal cortex; Mety: metyrapone.

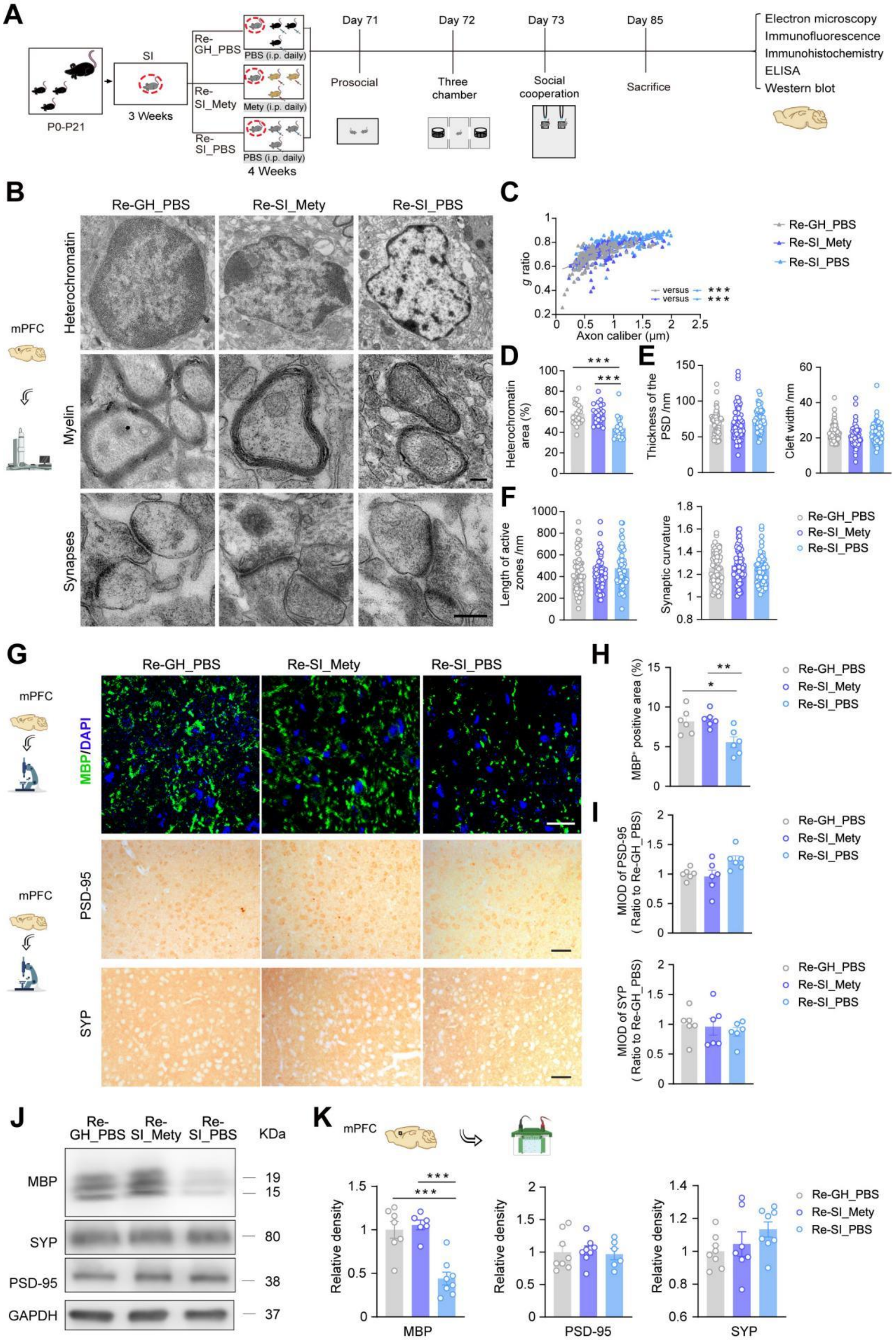
A
P0-P21
SI
3 Weeks
Re-GH_PBS
PBS (i.p. daily)
Re-SI_Mety
Mety (i.p. daily)
Re-SI_PBS
PBS (i.p. daily)
4 Weeks
Day 71
Prosocial
Day 72
Three chamber
Day 73
Social cooperation
Day 85
Sacrifice
Electron microscopy
Immunofluorescence
Immunohistochemistry
ELISA
Western blot
B
Re-GH_PBS
Re-SI_Mety
Re-SI_PBS
mPFC
Heterochromatin
Myelin
Synapses
C
g ratio
Axon caliber (μm)
versus ***
versus ***
D
Heterochromatin area (%)
E
Thickness of the PSD /nm
Cleft width /nm
F
Length of active zones /nm
Synaptic curvature
G
MBP/DAPI
PSD-95
SYP
H
MBP+ positive area (%)
I
MIOD of PSD-95 (Ratio to Re-GH_PBS)
MIOD of SYP (Ratio to Re-GH_PBS)
J
KDa
MBP
SYP
PSD-95
GAPDH
19
15
80
38
37
K
mPFC
Relative density
MBP
PSD-95
SYP

Fig. S10 SI companions treated with Mety improved the myelin dysfunction in SI mice during resocialization. **A** The timeline of the experimental design. **B** Representative EM images showing OLs nuclear heterochromatin, myelin, and synaptic morphology in the mPFC of mice. Scale bars, 500 nm. **C** Scatter plot of *g*-ratios with linear least squares fitting (Re-GH_PBS: 142 axons; Re-SI_Mety: 131 axons; Re-SI_PBS: 155 axons) (n = 6). **D** Graph showing the area percentage of OLs nuclear heterochromatin (Re-GH_PBS: 21 nuclei; Re-SI_Mety: 22 nuclei; Re-SI_PBS: 21 nuclei) (n = 6). **E, F** Bar graphs showing quantification of PSD, synaptic cleft width, length of the active zones, and synaptic curvature (Re-GH_PBS: 63 synapses; Re-SI_Mety: 63 synapses; Re-SI_PBS: 62 synapses). **G–I** Representative immunohistochemical images showing MBP (green), PSD-95, and SYP expression in the mPFC of mice (n = 6). **J, K** Representative bands and corresponding graphs showing MBP (both molecular weights), SYP, and PSD-95 protein expression in the mPFC of mice (n = 6). Data are presented as the mean ± SEM.*P <0.05, **P <0.01, ***P <0.001. Data were analyzed by one-way ANOVA followed by Tukey's *post hoc* test. GH: group housing; Re: resocialization; SI: social isolation; mPFC: medial prefrontal cortex; Mety: metyrapone.

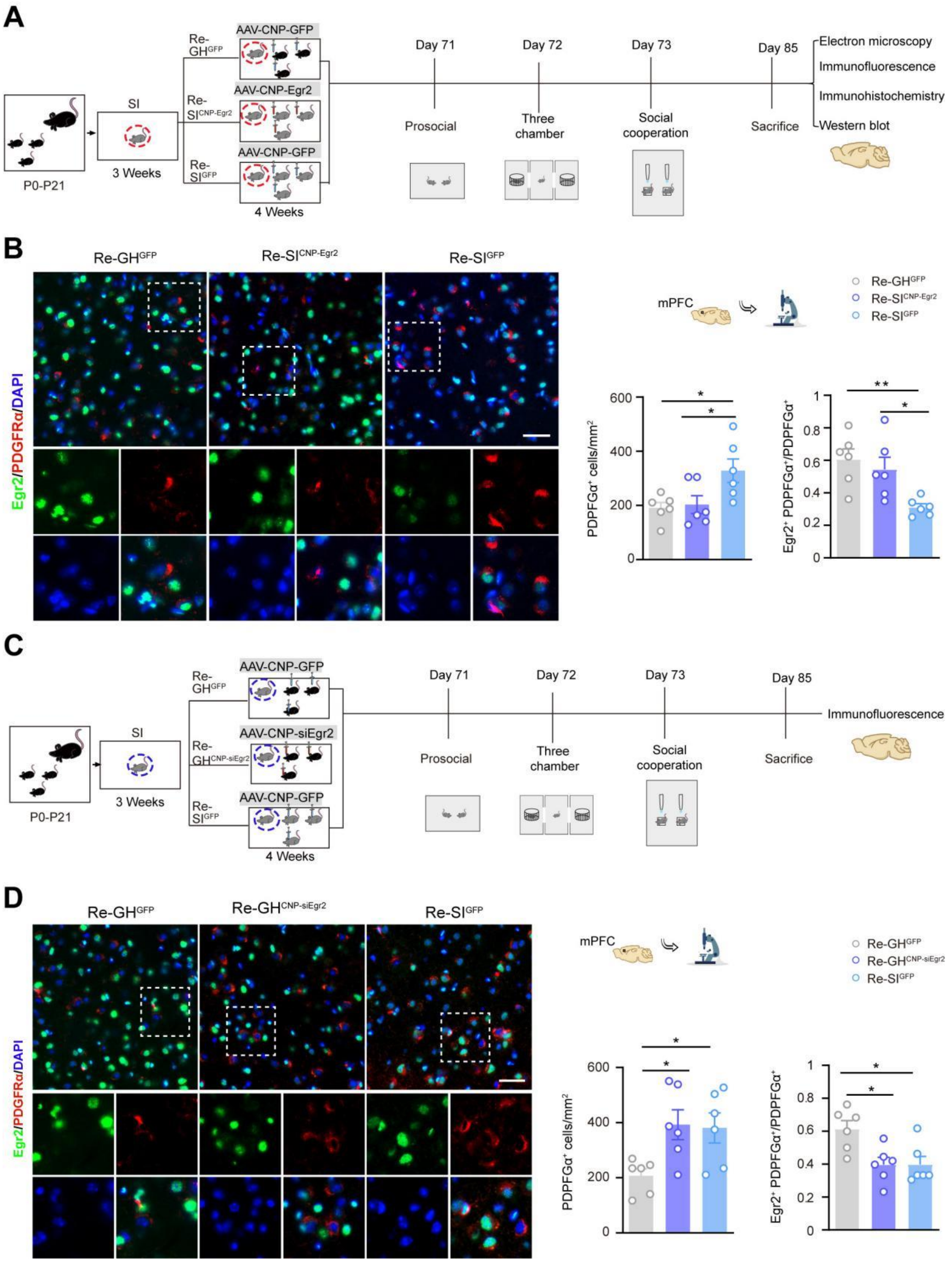

Fig. S11 Changes in PDGFRα expression in the mPFC of SI mice following co-housing with SI partners overexpressing Egr2 or GH partners down-regulating Egr2. **A, B** Representative

immunofluorescence images and corresponding graph showing PDGFRα^{+} (red) and Egr2^{+} (green) PDGFRα^{+}/PDGFRα^{+} expression in the mPFC of SI mice overexpressing Egr2 in mPFC OLs in the model of resocialization with other SI mice (*n* = 6). Scale bar, 50 μm. **C, D** Representative immunofluorescence images and corresponding graph showing PDGFRα^{+} (red) and Egr2^{+} (green) PDGFRα^{+}/PDGFRα^{+} expression in the mPFC of GH mice down-regulating Egr2 in mPFC OLs in the model of resocialization with other GH mice (*n* = 6). Scale bar, 50 μm. Data are presented as the mean ± SEM. *P <0.05, **P <0.01. Data were analyzed by one-way ANOVA followed by Tukey's *post hoc* test. mPFC: medial prefrontal cortex; GH: group housing; Re: resocialization; SI: social isolation.

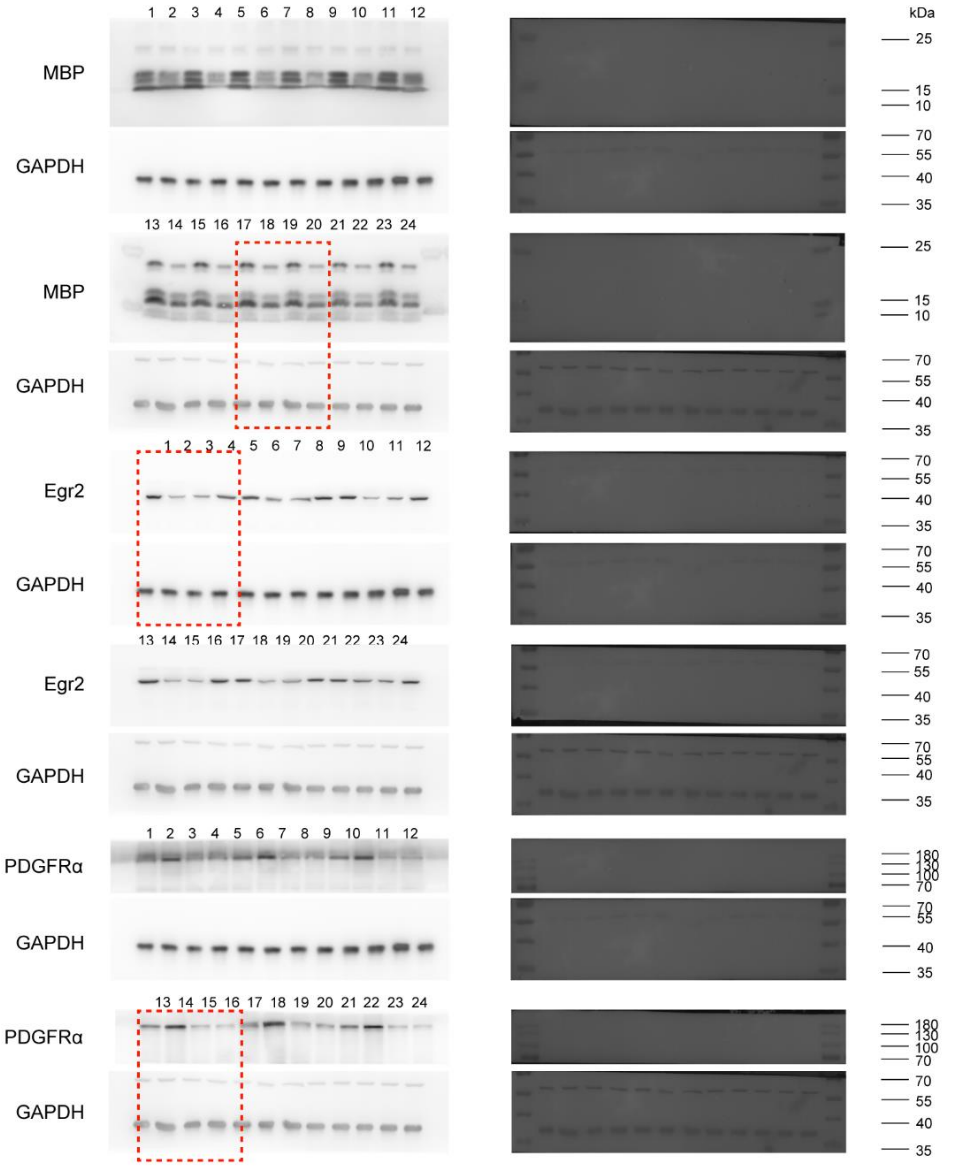

Con: 1, 5, 9, 13, 17, 21; siEgr2: 2, 6, 10, 14, 18, 22; siEgr2+siPDGFRα: 3, 7, 11, 15, 19, 23; siPDGFRα: 4, 8, 12, 16, 20, 24;

Uncropped western blot images. Uncropped blots are show for Fig. 5C.

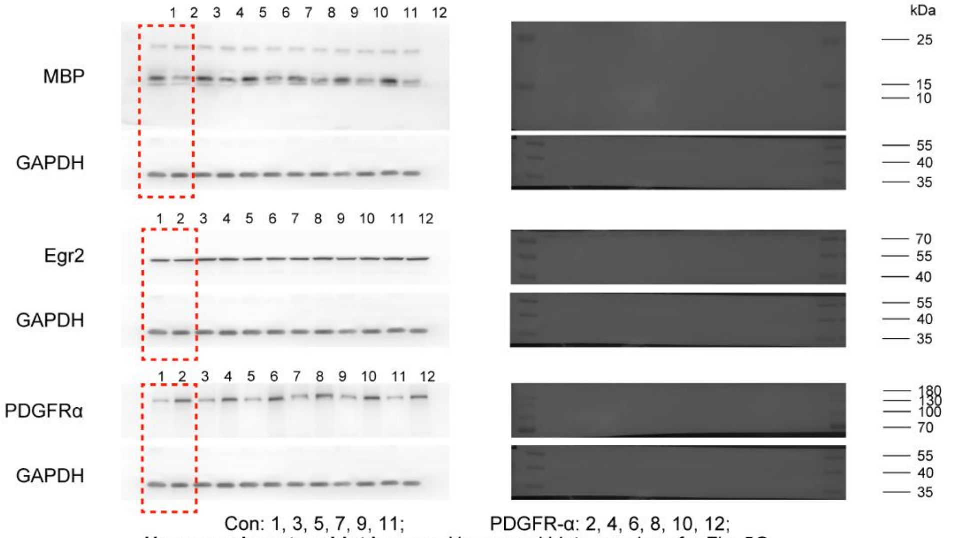

Con: 1, 3, 5, 7, 9, 11; PDGFR-α: 2, 4, 6, 8, 10, 12;

Uncropped western blot images. Uncropped blots are show for Fig. 5G.

Fig. S12 Uncropped blots for cropped images shown in Fig. 5C, G.

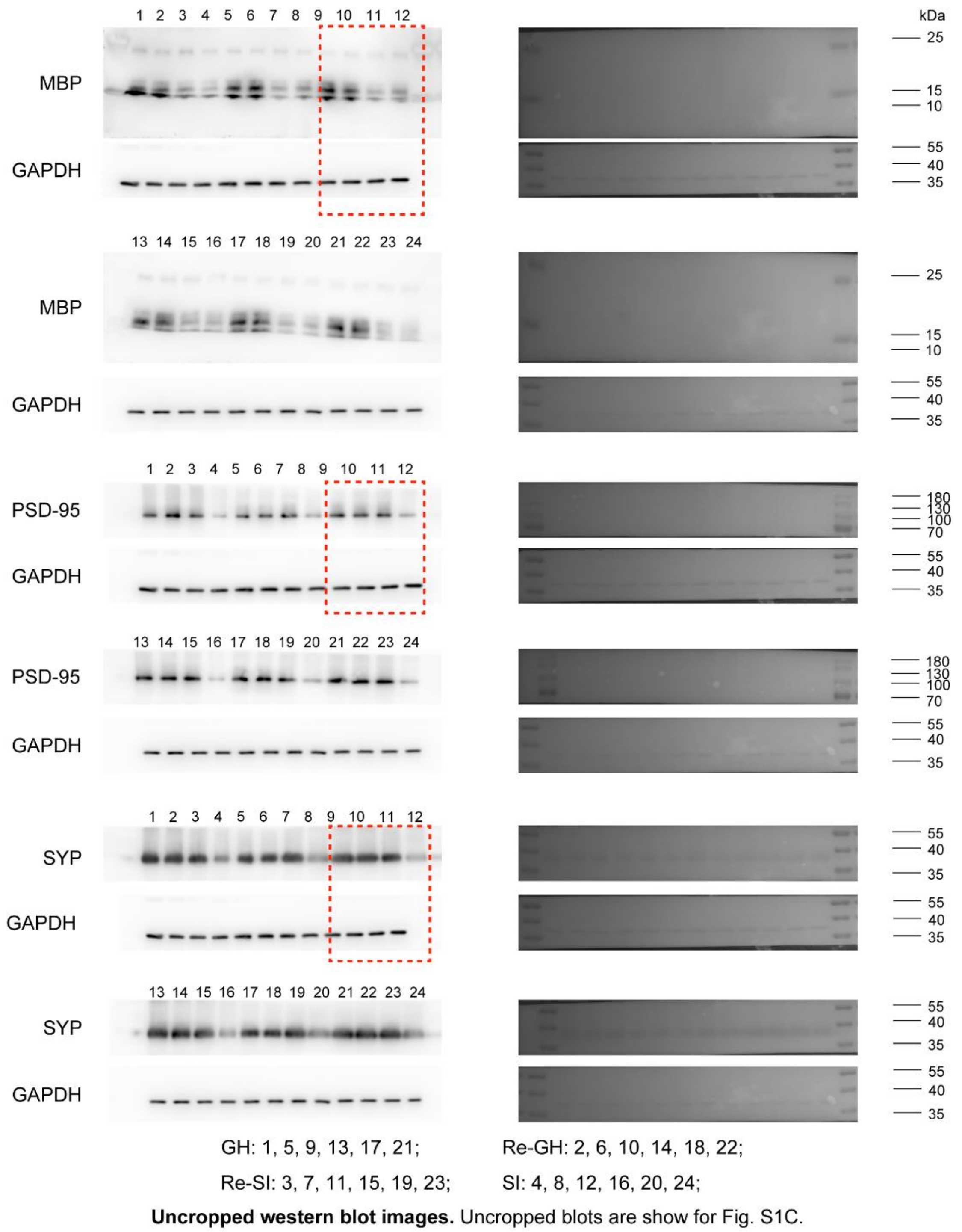

Fig. S13 Uncropped blots for cropped images shown in Fig. S1C.

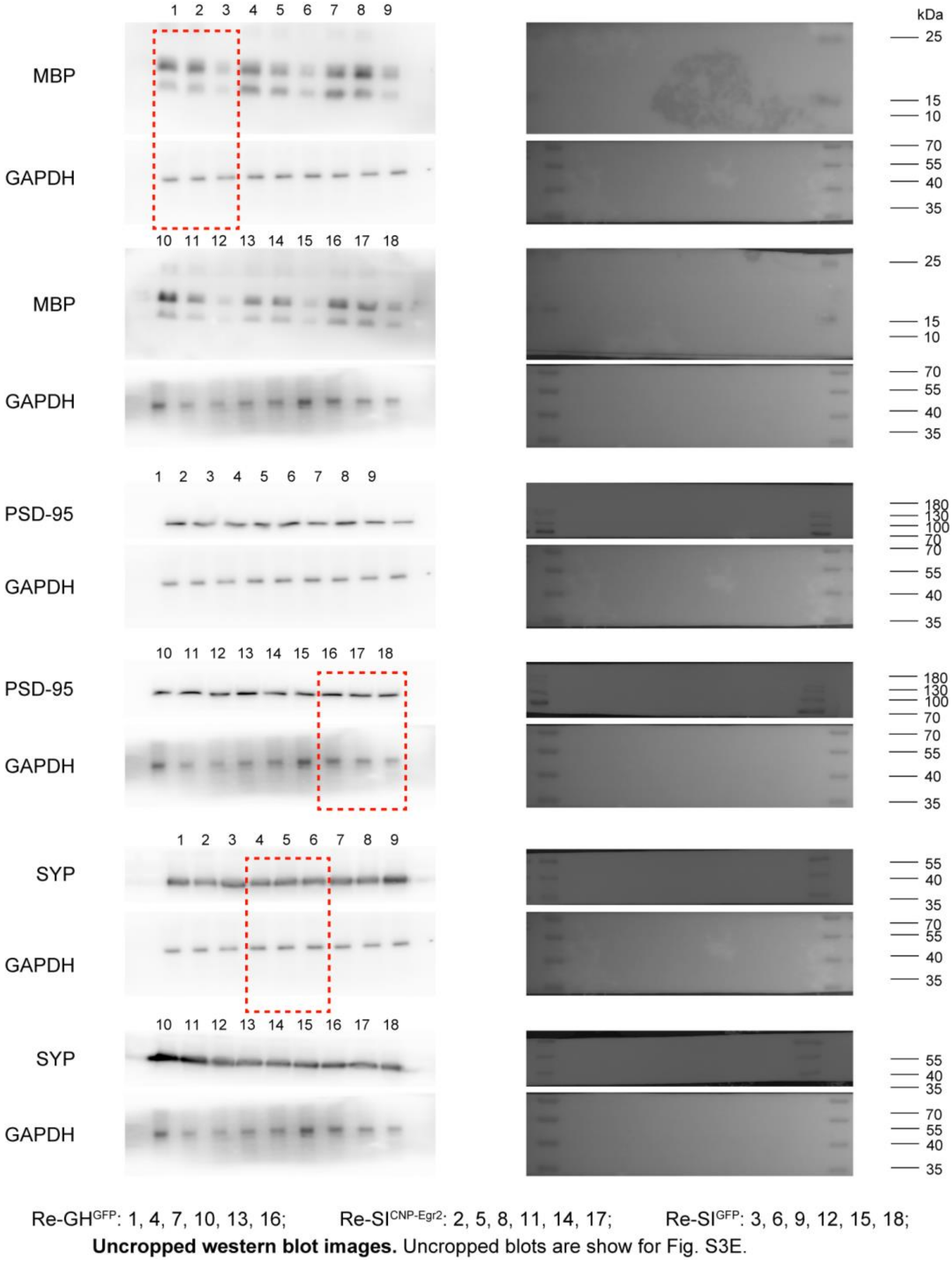

Fig. S14 Uncropped blots for cropped images shown in Fig. S3E.

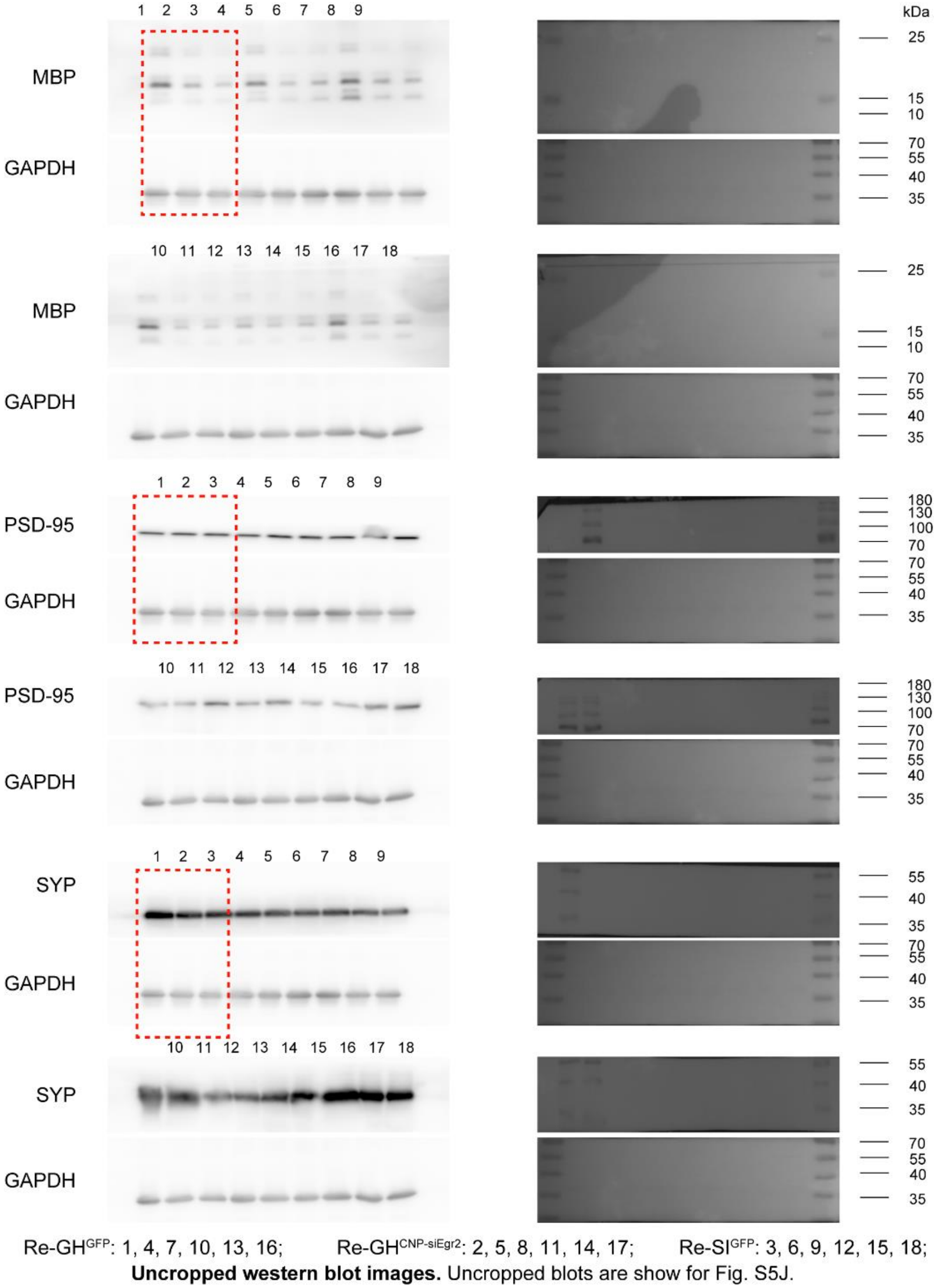

Fig. S15 Uncropped blots for cropped images shown in Fig. S5J.

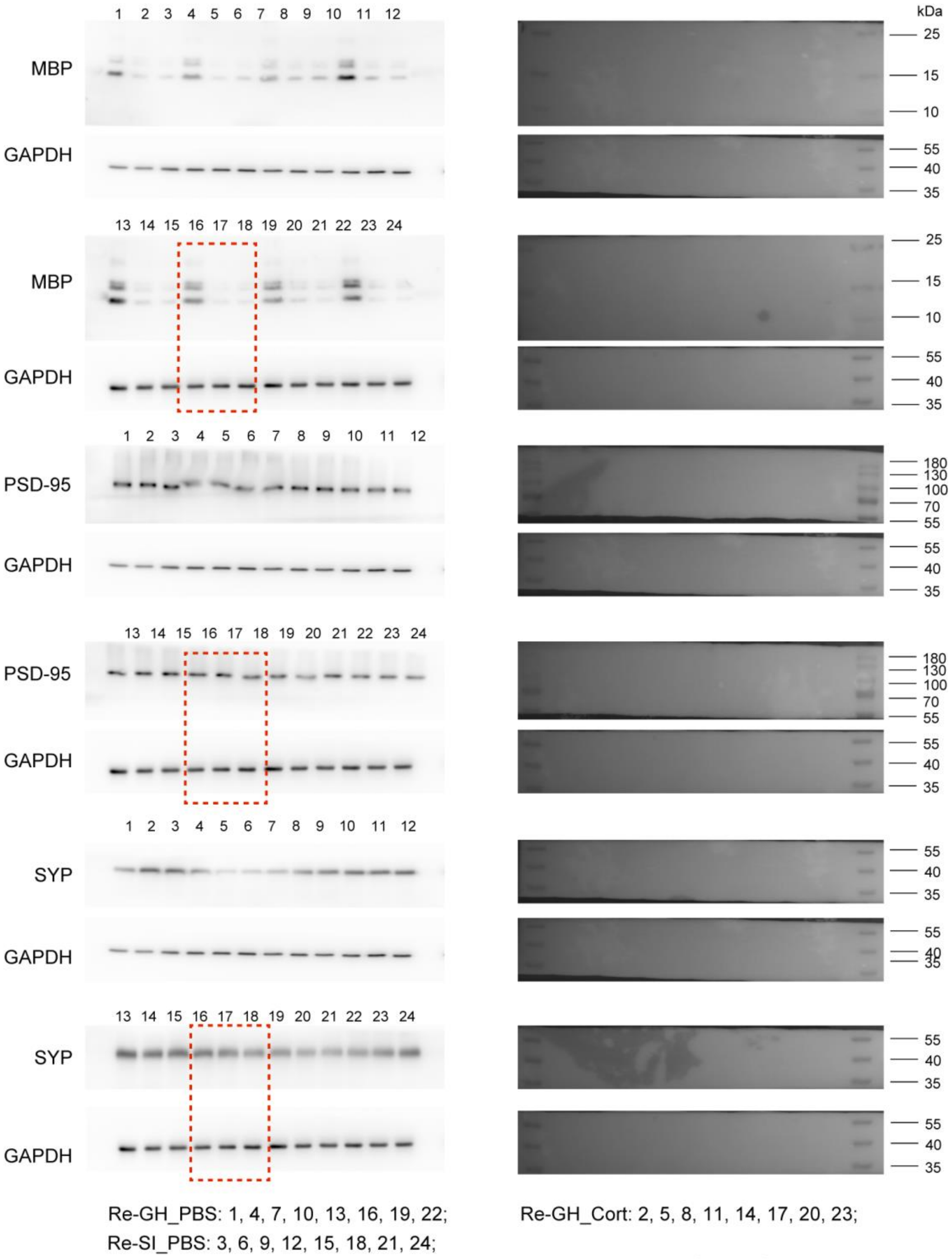

Uncropped western blot images. Uncropped blots are show for Fig. S8E.

Fig. S16 Uncropped blots for cropped images shown in Fig. S8E.

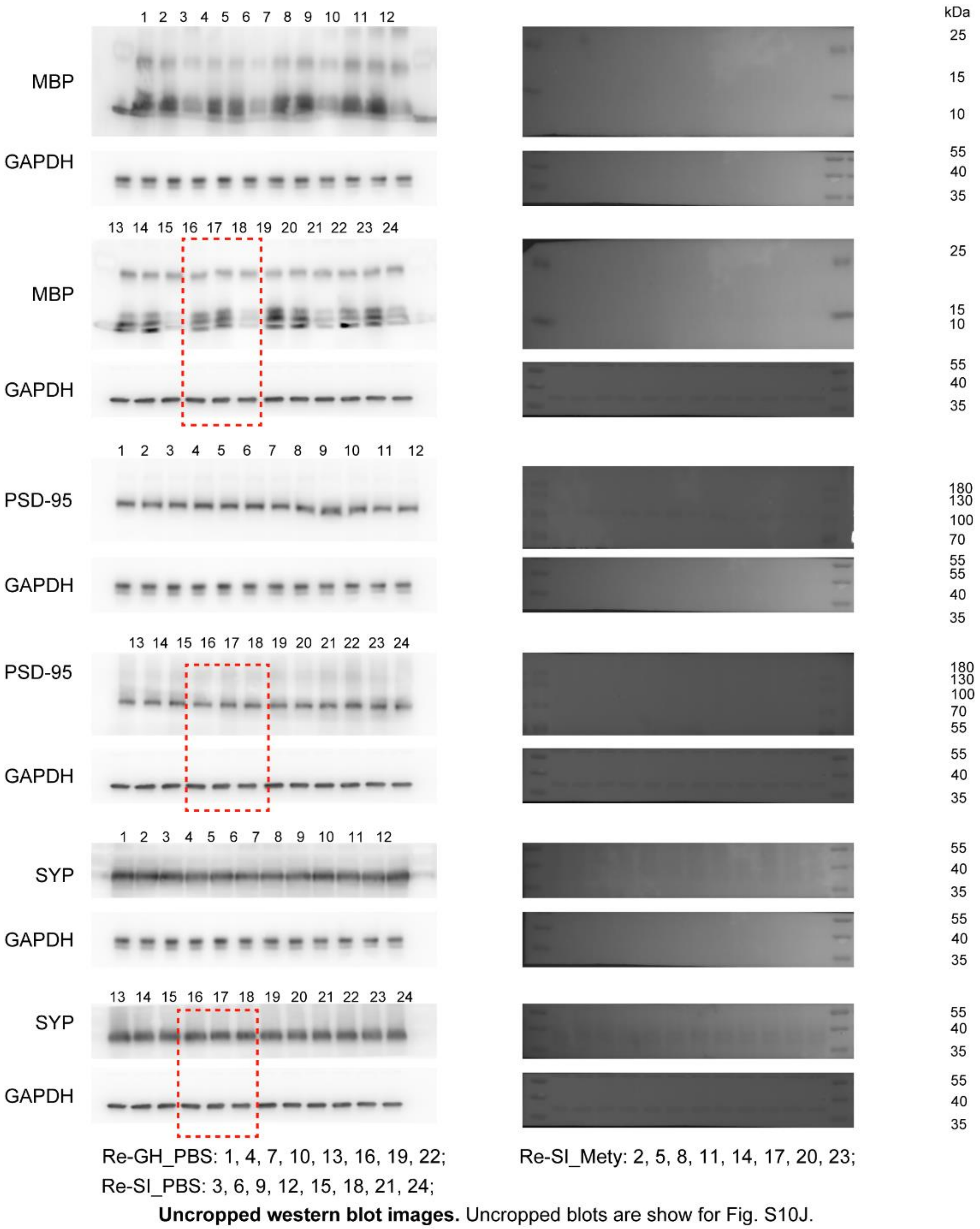

Fig. S17 Uncropped blots for cropped images shown in Fig. S10J.

Supplementary Tables

Table S1 Information on quantitative real-time PCR primers

Gene Symbol	Sequence (5' to 3')
Serpinb5-Forward	AACTGGAAGAAACGAAAGGTCAA
Serpinb5-Reverse	AGTAGGCAGCATTAACCACAAG
Mmp3-Forward	GGCCTGGAACAGTCTTGGC
Mmp3-Reverse	TGTCCATCGTTCATCATCGTCA
Smad3-Forward	AGGGGCTCCCTCACGTTATC
Smad3-Reverse	CATGGCCCGTAATTCATGGTG
Tgfb2-Forward	CTTCGACGTGACAGACGCT
Tgfb2-Reverse	GCAGGGGCAGTGTAAACTTATT
Gfap-Forward	CGGAGACGCATCACCTCTG
Gfap-Reverse	TGGAGGAGTCATTCGAGACAA
Pdgfra-Forward	TATCCTCCCAAACGAGAATGAGA
Pdgfra-Reverse	GTGGTTGTAGTAGCAAGTGTACC
Nfkb2-Forward	TGGCATCCCCGAATATGATGA
Nfkb2-Reverse	TGACAGTAGGATAGGTCTTCCG
Tnfrsf1a-Forward	GGGGATACATCCATCAGGGGT
Tnfrsf1a-Reverse	GCTCGGACAGTCACTCACC
IL-6-Forward	GGAGCCCACCAAGAACGATA
IL-6-Reverse	AGACAGGTCTGTTGGGAGTG
Tnf-Forward	CAGGCGGTGCCTATGTCTC
Tnf-Reverse	CGATCACCCCGAAGTTCAGTAG
Tgfb1-Forward	TACCAGAAATACAGCAACA
Tgfb1-Reverse	TGACATCAAAAGATAACCA
GAPGH-Forward	AGGTCGGTGTGAACGGATTTG
GAPGH-Reverse	GGGGTCGTTGATGGCAACA

Table S2 Information on quantitative real-time ChIP-PCR primers

Gene Symbol	Sequence (5' to 3')
0-150-Forward	CAATGAGAAAGGCTAGGCGTTG
0-150-Reverse	GGTCCCCGTGAACTTTTGATAATC
100-250-Forward	TTAGCGTCTCTTCCTTCTCCTGTA
100-250-Reverse	AACTCGCCCGAGGTTGG
200-350-Forward	GTATGATTCCCCAAAGTTCCCAAC
200-350-Reverse	GTCAACCCTTTGGATCTTTTTCCC
300-450-Forward	CAGGGAAAAAGATCCAAAGGGTTG
300-450-Reverse	ACTTGGTGAGCCTAACGGAATC
400-550-Forward	GACAGAAGTTCAGAAGGGGAGAAA
400-550-Reverse	CTTCCAGATTGCCCCCTCAAC
500-650-Forward	GGAAGGATTTTAGAACAGGGCAAG
500-650-Reverse	CTCTGGGGGCTGAAGGAAAG
600-750-Forward	CAGACCGGACCTTTCCTTCAG
600-750-Reverse	CTCAAGCGGTTGGTTTCTGAAAC
710-860-Forward	GTTTCAGAAACCAACCGCTTGAG
710-860-Reverse	CAAGCAAATGGTTGGGCAAAGATC
800-950-Forward	GATCTTTGCCCAACCATTTGCTTG
800-950-Reverse	TTCCATGTGTGACATTCCCTAAGG
900-1050-Forward	GGGAATGTCACACATGGAAACC
900-1050-Reverse	AGTATCGAGCACCCAGTTGC
1000-1150-Forward	CGATACTCATCCTTTTCTCCCCTT
1000-1150-Reverse	CTTCACAGTTCATTTCAGAGCCG
1100-1250-Forward	GGACTCCTTTAAAACGGCTCTGAA
1100-1250-Reverse	CTACCAGCTCTAATCCCACTACCT
1200-1350-Forward	TAGGGTGTTCCTTGCAAAGCAG
1200-1350-Reverse	GGGGGCTTTCATCTGTTGTTAA

1300-1450-Forward	TTAACAACAGATGAAAGCCCCC
1300-1450-Reverse	GCTAGCAGGATACCGTAATGTTTG
1400-1550-Forward	CAAACATTACGGTATCCTGCTAGC
1400-1550-Reverse	CCCTGCTCACTATTACTTCCACAT
1500-1650-Forward	GTAATAGTGAGCAGGGCAGTCAC
1500-1650-Reverse	GATATCAGGCTCGGGAAGAGGAT
1600-1750-Forward	CTCTTCCCGAGCCTGATATCC
1600-1750-Reverse	ACTATAGTGGACCTGAGGCTCTG
1700-1850-Forward	CGTTTTGTTATCAACCTGGTCACC
1700-1850-Reverse	CTCTATCTCCACCACCACCA
1800-1950-Forward	GTGAGATGGAGGTGGTGGTG
1800-1950-Reverse	CCTTCGACTTGCGCTCTATTTTTG
1850-2000-Forward	CACACACACACACCAAAAGCATTC
1850-2000-Reverse	CACACACACACACCAAAAGCATTC
